# USP10 Inhibits Ferroptosis via Deubiquinating POLR2A in Head and Neck Squamous Cell Carcinoma

**DOI:** 10.1002/advs.202412271

**Published:** 2025-07-02

**Authors:** Diekuo Zhang, Xueying Wang, Shanhong Lu, Yan Gao, Gangcai Zhu, Guo Li, Zongnan Yu, Junwei Hou, Helei Yan, Wenhui Yuan, Xin Zhang, Mien‐Chie Hung, Zhifeng Liu, Yong Liu

**Affiliations:** ^1^ Department of Otolaryngology Head and Neck Surgery Xiangya Hospital Central South University Changsha Hunan 410008 China; ^2^ Otolaryngology Major Disease Research Key Laboratory of Hunan Province 87 Xiangya Road Changsha Hunan 410008 China; ^3^ Clinical Research Center for Laryngopharyngeal and Voice Disorders in Hunan Province Changsha Hunan 410008 China; ^4^ National Clinical Research Center for Geriatric Disorders (Xiangya Hospital) Changsha Hunan 410008 China; ^5^ Xiangya Cancer Center Xiangya Hospital Central South University Changsha Hunan 410008 China; ^6^ Center for Molecular Oncology and Immunology Xiangya Hospital Central South University Changsha Hunan 410008 China; ^7^ Institute of Biochemistry and Molecular Biology China Medical University Taichung 406040 Taiwan; ^8^ Graduate Institute of Biomedical Sciences Research Center for Cancer Biology and Center for Molecular Medicine China Medical University Taichung 404328 Taiwan; ^9^ The First Affiliated Hospital Department of Otorhinolaryngology Hengyang Medical School University of South China Hengyang Hunan 421001 China

**Keywords:** ferroptosis, head and neck squamous cell carcinoma, POLR2A, SLC7A11, USP10

## Abstract

Ferroptosis has become a new way to induce cell death in cancer therapy. Deubiquitinating enzymes (DUBs) contribute to cancer ferroptosis, but underlying mechanisms are not completely understood. Here, it is discovered that USP10 as a member of DUBs, is tightly associated with a poor prognosis in patients with head and neck squamous cell carcinoma (HNSCC). Functionally, USP10 inhibits ferroptosis via transcriptionally upregulating the expression of *SLC7A11* in HNSCC. Targeting USP10 via gene depletion and antagonist sensitizes HNSCC cells to ferroptosis inducers both in vitro and in vivo. Mechanistically, USP10 directly interacts with the largest RNA Polymerase II Subunit A (POLR2A), removes the K48‐ and K63‐linked ubiquitin chains of POLR2A through its deubiquitinase activity and prevents ubiquitin‐mediated degradation. Then, POLR2A transcriptionally activates *SLC7A11*, eventually leading to the suppression of ferroptosis. Overall, the study indicates that a novel USP10‐POLR2A‐SLC7A11 axis regulates ferroptosis, positioning USP10 as a potential therapeutic target in patients with HNSCC.

## Graphical abstract

This study identifies USP10 as a novel deubiquitinase that antagonizes ferroptosis in head and neck squamous cell carcinoma (HNSCC). Mechanistically, USP10 directly stabilizes POLR2A protein through post‐translational deubiquitination, enabling POLR2A‐mediated transcriptional activation of SLC7A11, a key ferroptosis inhibitor. These findings verify a novel USP10‐POLR2A‐SLC7A11 axis as a therapeutically actionable pathway with clinical relevance in HNSCC.

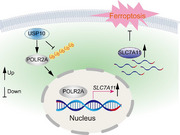

## 1 Introduction

Head and neck squamous cell carcinoma (HNSCC) ranks as the sixth frequent cancer type, with more than 600 000 new cases and over 300 000 deaths annually worldwide.^[^ ^1^ ^]^ HNSCC commonly arises from the oral cavity, pharynx, larynx etc. Patients with HNSCC are usually treated with surgery, radiation, chemotherapy, targeted therapy, immunotherapy, and a combination of these treatments. Treatment strategy for individual patient always depends on primary tumor location, cancer clinical stage, patient's requirements, age, and general health status etc.^[^ ^2^ ^]^ Although treatment efficacy has been significantly improved, part of patients with HNSCC are still refractory to current treatments and display a disappointing clinical response.^[^ ^2^ ^]^ Hence, identification of novel predictors and molecular targets is necessary and urgent to further improve the outcomes in these patient populations.

Ferroptosis refers to an iron‐dependent form of regulated cell death, which is stimulated by an overload of lipid peroxides on cellular membranes under diverse physiological conditions or pathological stresses.^[^ ^3^ ^]^ With canonical morphological and mechanistical characteristics that are different from other types of cell death including apoptosis, autophagy, necroptosis and pyroptosis, the roles of ferroptosis in cancer malignant behaviors and its effects on cancer immunity have been extensively investigated, highlighting ferroptosis as a promising therapeutic choice for the treatment of cancers that are resistant to existing conventional therapies.^[^ ^4^, ^5^ ^]^ At the morphological level, ferroptotic cancer cells display special features, such as shrunken mitochondria and reduction of mitochondrial cristae. Whereas mechanistically, functional disorders of tumor suppressors or oncogenes lead to unbalance of ferroptosis promotion systems (polyunsaturated fatty/PUFA synthesis and peroxidation, iron metabolism, and mitochondrial metabolism) and/or ferroptosis defense systems (GPX4‐GSH system, FSP1‐CoQH2 system, and DHODH‐CoQH2 system), which renders cancer cells vulnerable or resistant to ferroptosis.^[^ ^5^, ^6^ ^]^

As a highly conserved, reversible post‐translational modification and the tagging of proteolytic protein degradation, ubiquitination is well coordinated by ubiquitin‐activating (E1), ‐conjugating (E2), ‐ligating (E3) enzymes, and deubiquitinating enzymes (DUBs). Among these enzymes, DUBs cleave and remove the ubiquitin chains from protein substrates and reverse the function of ubiquitinase, hence stabilize target proteins.^[^ ^7^ ^]^ To date, DUBs have been extensively investigated as potential drug targets, due to their roles in the stabilization of key disease‐driving proteins, particularly in oncologic targets.^[^ ^8^, ^9^ ^]^ Indeed, six subfamilies of DUBs have been identified in human proteome including ubiquitin‐specific proteases (USPs), JAMNs, OTUs, MJDs, MINDYs, and UCHs. USPs comprise the largest family of DUBs, with ≈55 members.^[^ ^10^ ^]^ Current evidence has demonstrated that USP5,^[^ ^11^ ^]^ USP7,^[^ ^12^, ^13^, ^14^ ^]^ USP8,^[^ ^15^ ^]^ USP11,^[^ ^16^, ^17^, ^18^ ^]^ and USP14^[^ ^19^ ^]^ are able to regulate ferroptosis via directly deubiquitinating defense proteins (e.g., GPX4 or SLC7A11), or indirectly modulating upstreaming regulators in distinct cell types and patho/physiological contexts.

As a member of USPs, USP10 is involved in cancer progression via the modulation of protein substrates in multiple cancer types.^[^ ^20^, ^21^, ^22^, ^23^, ^24^, ^25^, ^26^, ^27^, ^28^ ^]^ It is abnormally expressed in solid cancers, such as ovarian cancer, gastric carcinoma, colorectal cancer, prostate cancer, esophageal squamous cell carcinoma, hepatocellular carcinoma and glioblastoma etc. In addition, its overexpression or downregulation correlated with cancer prognosis depending on cancer types.^[^ ^20^, ^21^, ^22^, ^23^, ^24^, ^25^, ^26^, ^27^, ^28^ ^]^ However, its roles in HNSCC and cancer ferroptosis remain unclear.

In this study, we identify that USP10 as an oncogene is tightly associated with a poor clinical prognosis in patients with HNSCC. Functional studies indicate that USP10 inhibits ferroptosis via transcriptionally upregulation of *SLC7A11*. Mechanistically, USP10 interacts with the largest RNA Polymerase II Subunit A (POLR2A), mediates its deubiquitination and then delays its protein degradation, which in turn upregulates the expression of *SLC7A11*. Together, our findings uncover the USP10‐POLR2A‐SLC7A11 axis as a critical ferroptosis regulator, which possesses therapeutic potential and clinical value in patients with HNSCC.

## 2 Results

### 2.1 USP10 Expression and its Clinical Significance in HNSCC

**Figure 1:**
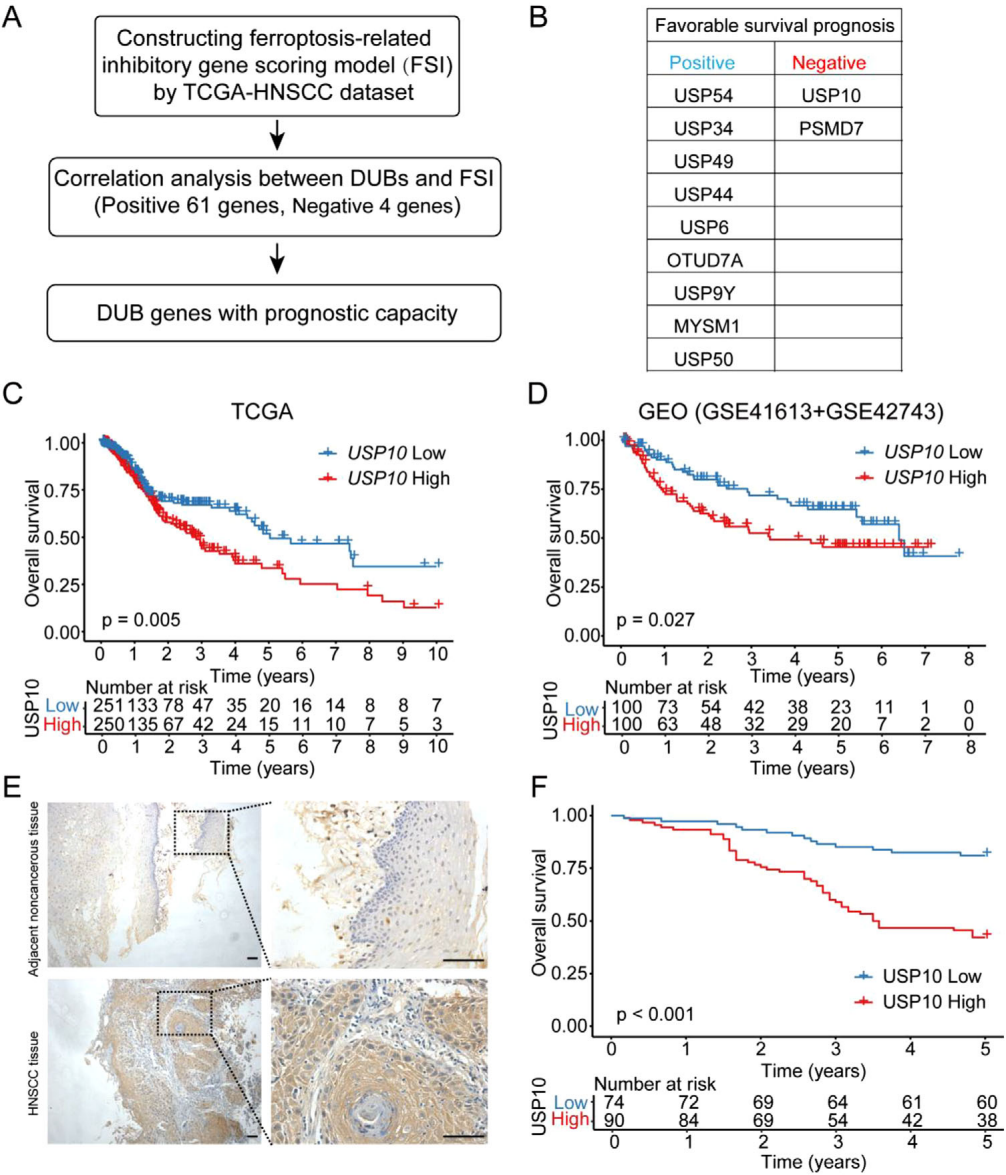
USP10 expression and its clinical significance in HNSCC. A) The schematic workflow of the study. B) Based on data from The Cancer Genome Atlas (TCGA), bioinformatic analysis identified 11 DUBs with prognostic capacity that are correlated with FSI. C,D) Kaplan–Meier curve analysis for correlations between *USP10* expression and overall survival of HNSCC patients in public online databases from TCGA (C, *n* = 501) and GEO merged by GSE41613 and GSE42743 (D, total *n* = 200). E) Representative immunohistochemical staining for USP10 protein in adjacent noncancerous epithelial tissues (upper) and HNSCC tissues (lower). Scale bars, 100 µm. F) Kaplan–Meier analysis of overall survival in all patients according to USP10 protein level. The log‐rank test was used to calculate the *p*‐value. FSI, Ferroptosis suppressor index.

DUBs and ferroptosis are involved in cancer development.^[^ ^29^ ^]^ To identify potential ferroptosis regulatory DUBs, 237 ferroptosis genes were used as the input gene set and scored to obtain a ferroptosis suppressor index (FSI) via the Cancer Genome Atlas (TCGA) public database. Then correlations between DUBs and FSI were analyzed via Spearman correlation analysis (**Figure** **1**A). As shown in Table S1 (Supporting Information), 61 DUBs were positively and 4 DUBs were negatively correlated with FSI. Gene levels of 11 DUBs were able to predict patients’ prognosis, in which high expression of *USP10* and *PSMD7* was tightly associated with an unfavorable prognosis in the TCGA patient cohort (Figure 1B,C; Figure S1A, Supporting Information). Additionally, two public online datasets of GSE42743 and GSE41613 (Table S2, Supporting Information) were further merged together to calculate the prognosis, which showed that only *USP10* (not *PSMD7*) possessed consistent prognostic capacity (Figure 1D; Figure S1B, Supporting Information), indicating its potential oncogenic role.

Due to cancer cell ferroptosis is closely associated with chemotherapy or radiotherapy,^[^ ^30^, ^31^ ^]^ patients with intact therapy and prognosis information in TCGA dataset were stratified to analyze the potential associations between USP10 and treatment modalities. We found that patients with USP10 high expression showed a worse prognosis in patients treated with single radiotherapy and radio‐plus‐chemotherapy, indicating that USP10 may contribute to efficiency of radiotherapy and chemotherapy (Figure S1C, Supporting Information). Inspired by the concept that inhibitors of oncogenic DUBs have been approved by the Food and Drug Administration (FDA), and widely applied in preclinical and clinical practice, therefore, our following study focused on *USP10*.

Next, clinical significance of USP10 protein was investigated in 167 samples of paraffin‐embedded, archival HNSCC primary samples via immunohistochemical staining. Our staining revealed that USP10 protein level was higher in HNSCC than that in corresponding adjacent noncancerous samples (Figure 1E). Importantly, USP10 protein was obviously correlated with multiple clinical parameters, such as primary tumor site, T classification, clinical stage, lymph node or distant metastasis (all *p* < 0.05; Table S3, Supporting Information). Kaplan–Meier survival calculation revealed that high expression of USP10 protein was negatively correlated with overall survival rates (Figure 1F). Both univariate and multivariate Cox regression analysis demonstrated that USP10 expression was an independent prognostic variable in patients with HNSCC (Table S4, Supporting Information).

### 2.2 USP10 Depletion Enhances Susceptibility to Ferroptosis In Vitro and In Vivo

**Figure 2:**
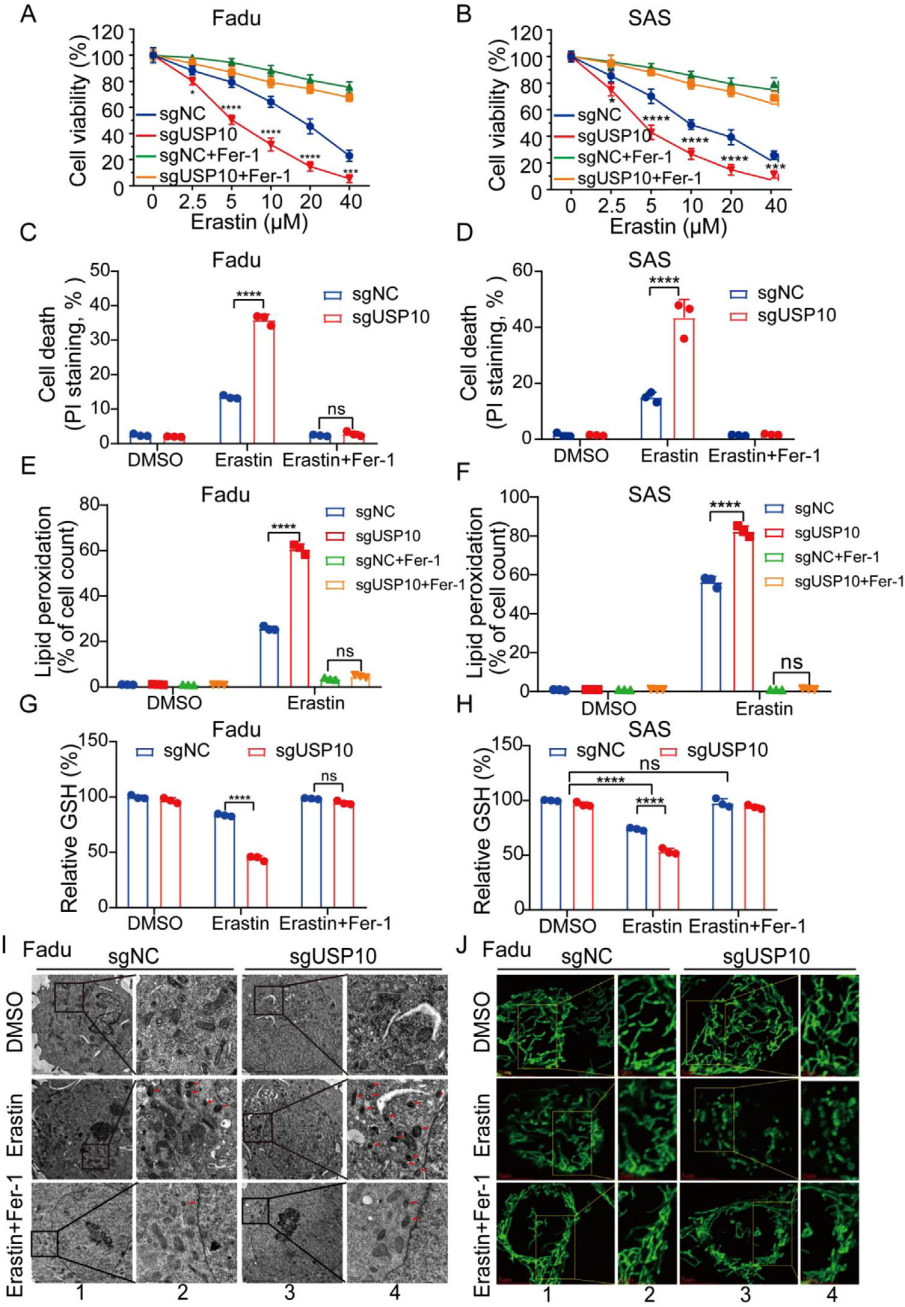
USP10 depletion enhances susceptibility to ferroptosis. A,B) USP10 stable depletion mediated by CRISPR‐Cas9 in Fadu and SAS cells that were treated with Erastin for 24 h, with or without the addition of classical ferroptosis inhibitor Fer‐1 (Ferrostatin‐1, 1 µm). Cell viability was assessed by CCK8 assays. C–H) Indicated Fadu and SAS cells were treated with 10 µm Erastin. Then cell death (C,D) was analyzed by PI staining, lipid peroxidation levels (E,F) were detected by flow cytometry after staining with 5 µm BODIPY‐C11 probes, GSH levels (G,H) were detected with GSH and GSSG assay kits. I,J) Fadu cells were treated with 10 µm Erastin, with or without the addition of Fer‐1, alterations of mitochondria were observed by transmission electron microscopy (TEM). Inner red arrows indicated canonical mitochondria in cells experiencing ferroptosis. Scale bars, 4 µm (columns 1 and 3) and 1 µm (columns 2 and 4). I). After the staining of Rho123, a known mitochondria‐tracking probe, mitochondria were analyzed by structured illumination microscopy (SIM). Scale bars, 5 µm (columns 1 and 3) and 2 µm (columns 2 and 4) J). All data are representative of at least three independent experiments. Data are presented as mean ± SD, *n* = 3. *p* value was determined by two‐way ANOVA (A,F) and two‐tailed unpaired Student's *t*‐test (G,H); ns, not significant (*p* > 0.05); * *p* < 0.05; ** *p* < 0.01; *** *p* < 0.001; **** *p* < 0.0001.

To directly investigate the role of USP10 in ferroptosis, HNSCC Fadu and SAS cell lines that highly expressed USP10, were used to generate stable USP10‐depleted cells via CRISPR for the following functional experiments (Figure S2A–F, Supporting Information). USP10 depletion increased the sensitivity to inducers of Erastin and RSL3 in Fadu and SAS cells in a dose‐dependent manner (**Figure** **2**A,B; Figure S3A,B, Supporting Information), suggesting that ferroptosis is involved in the process of cell death driven by USP10 depletion. Meantime, USP10 depletion dramatically increased cell death (Figure 2C,D; Figure S3C,D, Supporting Information), lipid peroxidation (Figure 2E,F; Figure S3E–I, Supporting Information), labile iron level (Figure S3J, Supporting Information) and reduced GSH level (Figure 2G,H; Figure S3K,L, Supporting Information) triggered by Erastin and RSL3. Transmission electron microscopy (TEM) confirmed that USP10‐depleted Fadu cells that exposed to Erastin, contained smaller, shrunken mitochondria (Figure 2I). Additionally, alteration in mitochondrial morphology was vividly visualized by the fluorescent staining with rhodamine (Rho123), a well‐known mitochondria tracing probe (Figure 2J), which well visualized the morphological signature of ferroptotic cells. As to mitochondrial potential, JC‐1 assays revealed that USP10 depletion dramatically decreased mitochondrial membrane potential following Erastin treatment (Figure S3M,N, Supporting Information).

Importantly, cell morphology, decreased cell viability, increased cell death, and lipid peroxidation triggered by Erastin and RSL3 in USP10‐depleted cells, could be prevented by ferroptosis inhibitor Ferrostatin‐1 (Fer‐1) in Fadu and SAS cells (Figure 2A–J; Figure S3C–L, Supporting Information). As controls, inhibitors of other types of cell death, including autophagy (3‐methylademine, 3‐MA) and apoptosis (Z‐VAD), failed to suppress cell death induced by Erastin in USP10‐depleted cells (Figure S4A–D, Supporting Information), suggesting that ferroptosis inducers‐triggered cell death is independent of autophagy and apoptosis.

**Figure 3:**
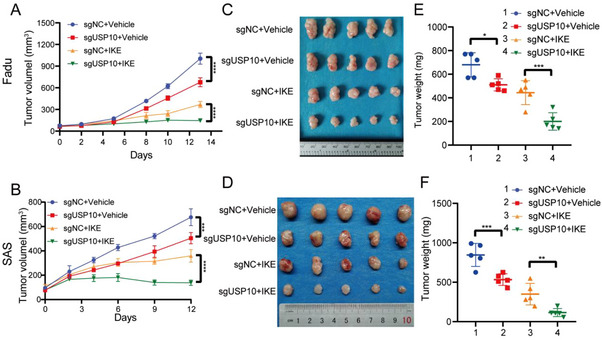
Targeting USP10 sensitizes HNSCC to ferroptosis in vivo. A–F) 2 × 10^6^ Fadu cells (A,C,E) and 1 × 10^6^ SAS cells (B,D,F) were injected subcutaneously into each nude mouse (*n* = 5 per group); when tumor volumes reached 80–90 mm^3^, 40 mg kg^−1^ IKE or vehicle was intraperitoneally administered daily for 13 days (Fadu cells) and 12 days (SAS cells). Growth curves calculated by tumor volume (A,B), harvested xenograft tumors (C,D) and tumor weight (E,F) were provided. All data are representative of at least three independent experiments. Data are presented as mean ± SD, *n*  =  5. *p* value was determined by two‐way ANOVA; ns, not significant (*p* > 0.05); * *p* < 0.05; ** *p* < 0.01; *** *p* < 0.001; **** *p* < 0.0001.

Simultaneously, in vivo experiments were further employed to consolidate the in vitro results. Our data indicated that USP10‐depleted Fadu and SAS cells grew slowly in nude mice. Combined with the usage of Imidazole ketone erastin (IKE, an Erastin analog with a more potent, selective and stable inducer of ferroptosis), an obviously synergistic tumor inhibitory effect was observed in mice treated by USP10 depletion plus IKE, which was manifested by the tumor growth pattern (**Figure** **3**A,B), harvested xenografted tumors (Figure 3C,D) and tumor weight (Figure 3E,F) and cell proliferation marker Ki67 and lipid peroxidation marker 4‐hydroxy‐2‐nonenal (4HNE) (Figure S5, Supporting Information). Taken together, these results clearly consolidate the notion that USP10 depletion sensitizes HNSCC cells to ferroptosis both in vitro and in vivo.

### 2.3 USP10 Blocks Cancer Ferroptosis Through Transcriptional Activation of *SLC7A11*

**Figure 4:**
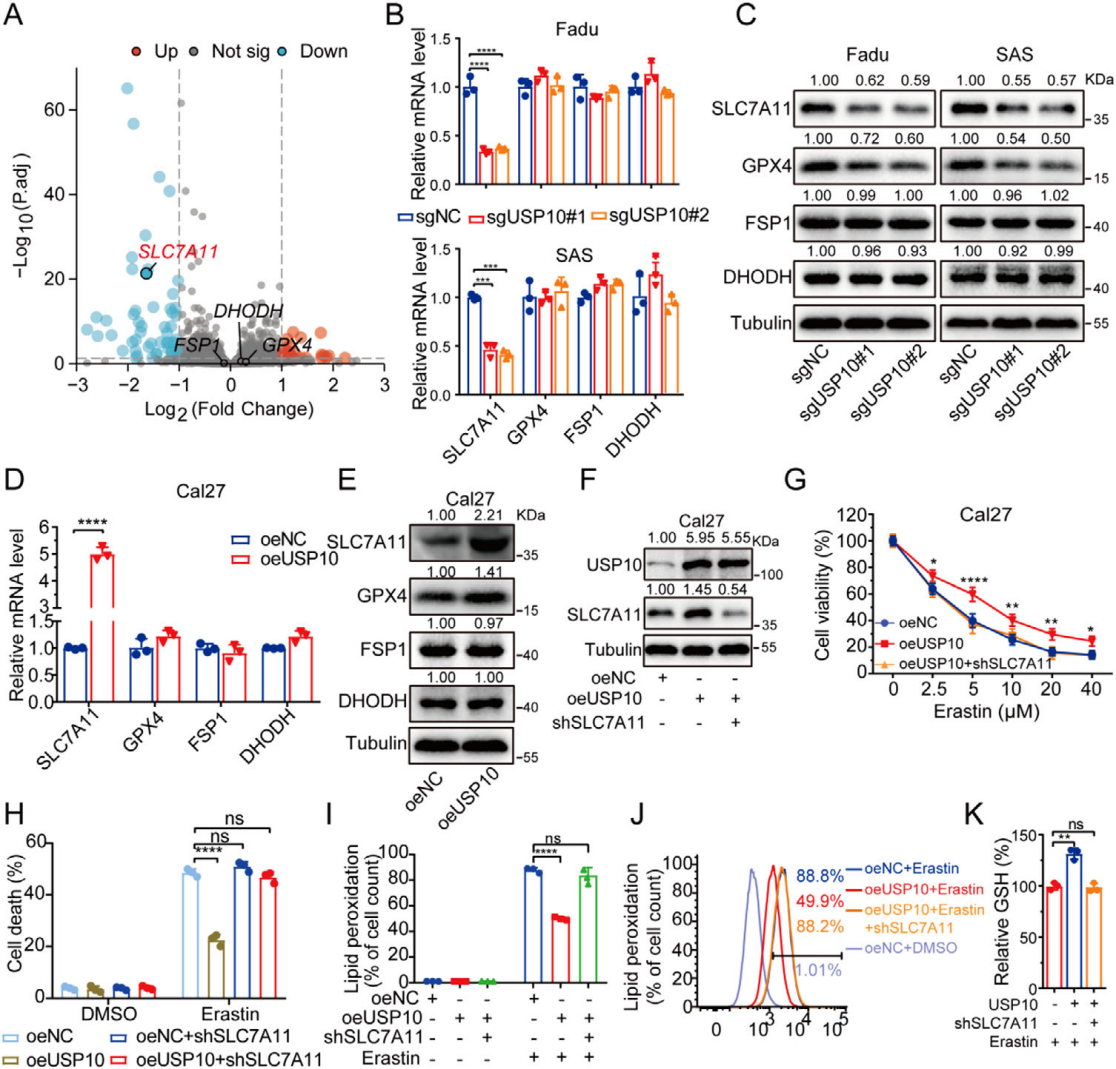
USP10 regulates ferroptosis sensitivity through SLC7A11. A) Volcanic plot showed the DEGs with 196 decreased (blue) and 262 increased (red) genes in USP10‐depleted Fadu cells. SLC7A11 highlighted in red. B–E) mRNA levels of *SLC7A11*, *GPX4*, *FSP1*, and *DHODH* were quantified by qPCR (B,D), and their corresponding proteins were checked by immunoblot assays (C,E) in USP10‐depleted (sg1, sg2) or overexpressed (oeUSP10) versus control HNSCC cells (sgNC and oeNC). F) Lentivirus‐mediated shRNA was used to inhibit SLC7A11 in USP10 overexpressed Cal27 cells and proteins were examined by immunoblot. G–K) Indicated cells were treated with Erastin and cell viability were assessed by CCK8 (G), cell death (H) and lipid peroxidation levels (I,J) were checked by flow cytometry with the staining of PI and BODIPY‐C11 probes, GSH levels (K) were detected with GSH and GSSG assay kits. Tubulin was used as a protein internal control (C,E,F). All data are representative of at least three independent experiments. Data are presented as mean ± SD, *n* = 3. *p* value was determined by two‐tailed unpaired Student's *t*‐test (B,D,K) and two‐way ANOVA (G,H,I); ns, not significant (*p* > 0.05); * *p* < 0.05; ** *p* < 0.01; *** *p* < 0.001; **** *p* < 0.0001.

There are three major cellular protective systems against ferroptosis including GPX4, FSP1, and DHODH.^[^ ^32^ ^]^ To clarify through which system USP10 regulates cancer ferroptosis, USP10‐depleted and control Fadu cells were subjected to RNA‐seq, which clearly showed that *SLC7A11*, an upstreaming regulator of GPX4, was significantly declined in UPS10‐depleted Fadu cells (**Figure** **4**A; Table S5, Supporting Information). qPCR assays validated that USP10 depletion led to a transcriptional inhibition of *SLC7A11*, while mRNA levels of *GPX4*, *FSP1*, and *DHODH* did not change in Fadu and SAS cells (Figure 4B). At the protein level, both SLC7A11 and its downstreaming GPX4 were diminished following USP10 depletion, whereas proteins of FSP1 and DHODH stayed unaltered (Figure 4C). In addition, USP10 overexpressed in HNSCC Cal27 cells (Figure 4D; Figure S6A,B, Supporting Information), and we found that *SLC7A11* mRNA, proteins of SLC7A11 and GPX4 were correspondingly elevated, while *GPX4* mRNA, mRNAs and proteins of FSP1 and DHODH still remained at the same level (Figure 4D,E). As a deubiquitinase, immunoprecipitation (IP) confirmed that USP10 did not connect to proteins of SLC7A11 and GPX4 (Figure S6C, Supporting Information). It has been reported that SLC7A11 is able to facilitate GPX4 protein synthesis in polysome,^[^ ^33^ ^]^ therefore, the results suggest that USP10 may transcriptionally activate *SLC7A11*.

To confirm whether SLC7A11 is involved in USP10‐mediated regulation of cancer ferroptosis, reversal assays were performed by transfecting the shSLC7A11 plasmid into the USP10‐overexpressed Cal27 cells (Figure 4F). As expected, USP10 overexpression inhibited the susceptibility to Erastin and RSL3, which was manifested by enhanced cell viability, declined cell death, decreased lipid peroxidation and elevated GSH. This effect could be totally reversed by ferroptosis inhibitor Fer‐1 (Figure S6D–H, Supporting Information). Following the inhibition of SLC7A11, USP10 mediated effects were correspondingly reversed (Figure 4G–K). Taken together, our data indicate that USP10 blocks cancer ferroptosis through the transcriptional activation of *SLC7A11*.

### 2.4 USP10 Elevates SLC7A11 Expression Through POLR2A

**Figure 5:**
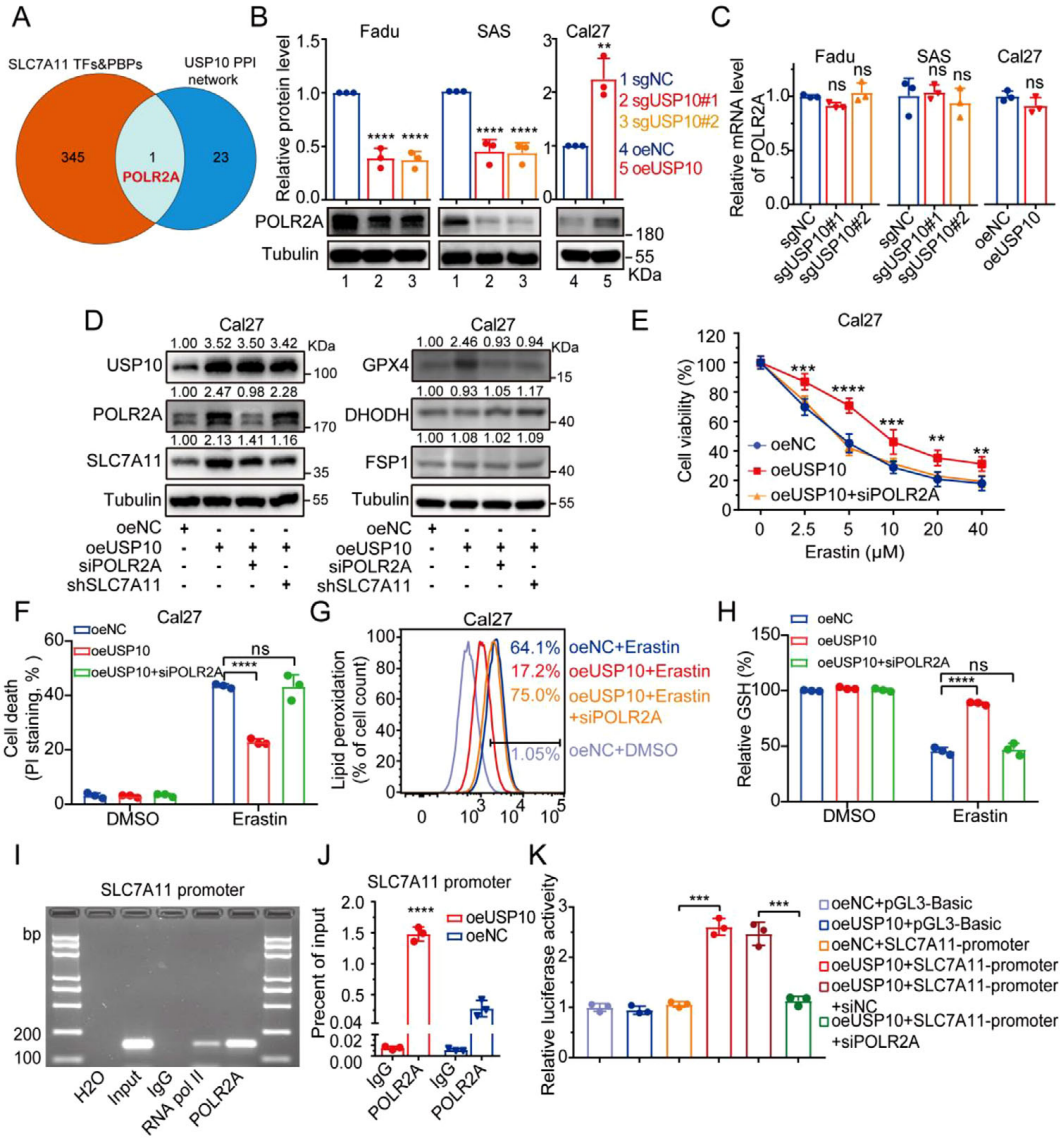
USP10 elevates SLC7A11 through increasing protein expression of POLR2A. A) The String database was used to predict the potential proteins that interact with USP10. Venn diagram of the transcription factors (TFs) and promoter binding proteins (PBPs) of *SLC7A11* (red) and interacting proteins of USP10 (blue) showing that POLR2A was bound to both USP10 and the promoter of *SLC7A11*. B,C) Protein and mRNA levels of POLR2A were quantified by immunoblot (B) and qPCR (C) in stated USP10‐depleted or overexpressed HNSCC cells. D) Immunoblot assays displayed protein levels in oeUSP Cal27 cells accompanied by the inhibition of POLR2A or SLC7A11. E–H) Cell viability (E) was checked by CCK8, cell death (F) and lipid peroxidation levels (G) were checked by flow cytometry with the staining of PI and BODIPY‐C11 probes, GSH levels (H) were detected with GSH and GSSG assay kits. I,J) Representative image of ChIP‐PCR analysis showing the connection between POLR2A and the promoter region of *SLC7A11*. K) Relative luciferase activity of *SLC7A11* promoter regions. PGL3‐basic is negative control. All data are representative of at least three independent experiments. Data are presented as mean ± SD, *n* = 3. *p* value was determined by two‐tailed unpaired Student's *t*‐test (B,C,J) and two‐way ANOVA (E,F,H,K); ns, not significant (*p* > 0.05); ** *p* < 0.01; *** *p* < 0.001; **** *p* < 0.0001.

To explore how USP10 activates *SLC7A11*, String (https://string‐db.org) was used to predict the interactome of USP10,^[^ ^34^ ^]^ UCSC database (https://genome.ucsc.edu) and JASPAR software were applied to obtain transcription factors (TFs) that bind to the promoter region of *SLC7A11*.^[^ ^35^ ^]^ As shown in **Figure** **5**A, 346 possible mutual interaction proteins with USP10, and 24 potential TFs of *SLC7A11* were identified, in which POLR2A was found to interact with USP10, and meantime could act as a potential TF that bind to the promoter region of *SLC7A11*. USP10 depletion resulted in regulated protein expression of POLR2A (Figure 5B), but no fluctuations of its mRNA in HNSCC cells (Figure 5C). Together, these data suggest that USP10 increases the protein level of POLR2A.

To answer the question whether POLR2A participates in the USP10‐medidated transcriptional activation of *SLC7A11* and inhibition of cancer ferroptosis, we knocked down the expression of POLR2A in USP10‐overexpressed Cal27 cells. Our results indicated that POLR2A inhibition hindered the protein elevation of SLC7A11 and GPX4, which was induced by USP10 overexpression, while FSP1 and DHODH protein levels were not affected by POLR2A inhibition (Figure 5D). Moreover, POLR2A knockdown reversed the effects caused by USP10 overexpression, including cell viability, cell death, lipid peroxidation, and GSH level (Figure 5E–H).

To further validate whether POLR2A is a true TF in USP10‐mediated transcription of *SLC7A11*, analysis via USUC database predicted that POLR2A may bind to *SLC7A11* promoter at the region of 5‐174 bp. Subsequently, chromatin immunoprecipitation (ChIP)‐PCR assays showed that chromatin fragments corresponding to this promoter region were specifically present in the anti‐POLR2A immunoprecipitates from Cal27 cells (Figure 5I). The binding of POLR2A to *SLC7A11* promoter was increased in USP10‐overexpressed Cal27 cells (Figure 5J). To verify that the potential *SLC7A11*‐binding site was indeed responsive to POLR2A, luciferase reporter plasmids were constructed and then these plasmids were individually transfected into USP10‐overexpressed Cal27 cells with or without POLR2A inhibition. As shown in Figure 5K, the luciferase activity in USP10‐overexpressed cells dramatically increased, and correspondingly declined following POLR2A inhibition. Together, our results support that USP10 upregulates the protein expression of POLR2A, which in turn binds to the promoter region of *SLC7A11* and activates its mRNA transcription.

### 2.5 USP10 Interacts with POLR2A

**Figure 6:**
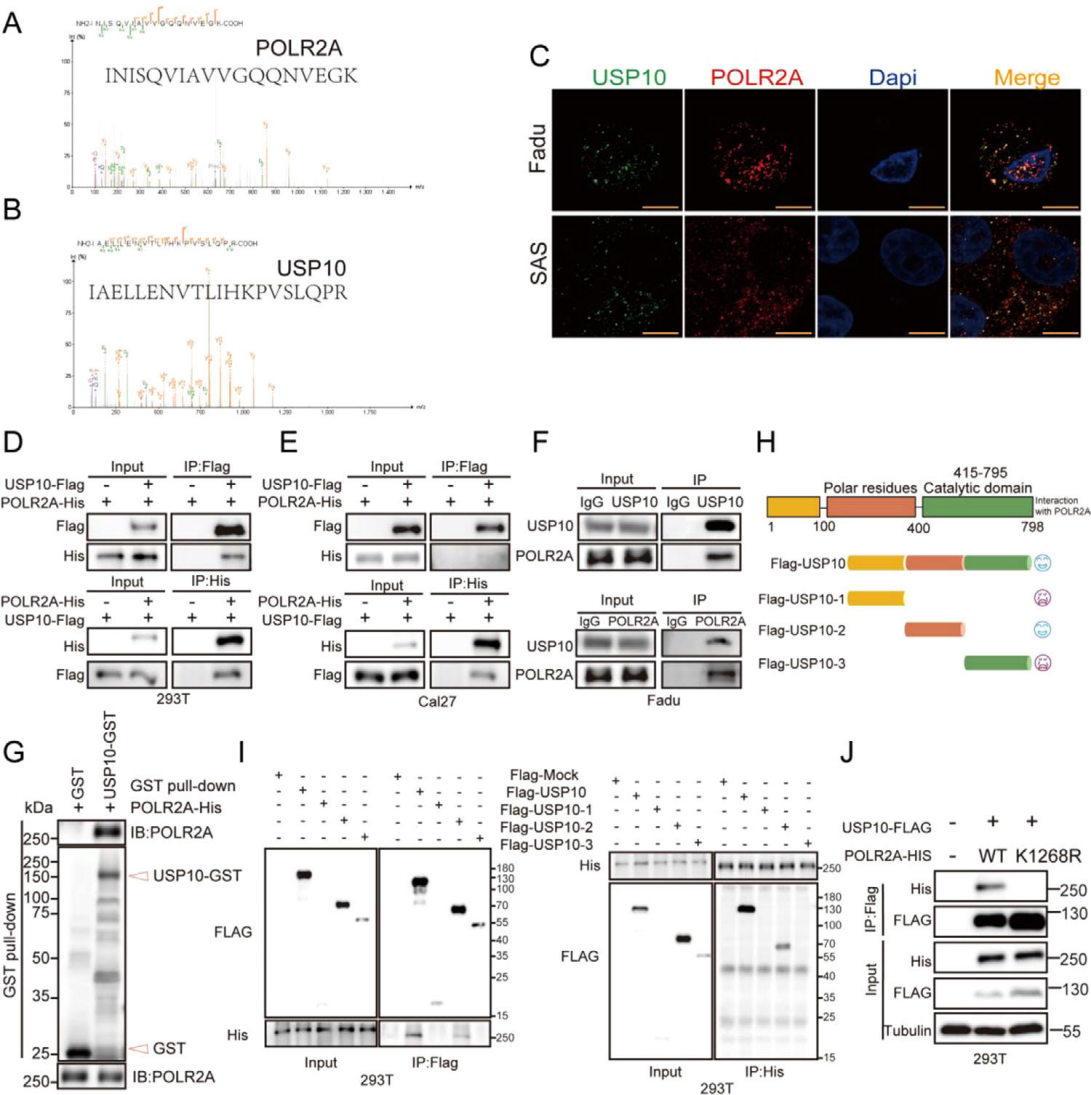
USP10 increases POLR2A expression and maintains its protein stability. A,B) MS/MS spectra showing doubly charged IAELLENVTLIHKPVSLQPR and INISQVIAVVGQQNVEGK peptides from USP10 and POLR2A, respectively. The single‐letter abbreviations for amino acid residues are as follows: A, Ala; D, Asp; E, Glu; G, Gly; H,His; I, Ile; K, Lys; L, Leu; N, Asn; P, Pro; Q, Gln; R, Arg; S, Ser; T, Thr; V, Val. m/z, mass/charge ratio; int, relative intensity. C) Representative confocal images showing the colocalization of USP10 (green) and POLR2A (red) in Fadu and SAS cells. Scale bars, 5 µm. D,E) The indicated plasmids were transfected into HEK293T or Cal27 cells for 48 h, then harvested with protein lysis buffer was use for co‐immunoprecipitation. F) Interaction of endogenous USP10 and POLR2A in Fadu cells was detected by co‐immunoprecipitation. G) Purified His‐POLR2A WT was incubated with GST or GST‐USP10 coupled with glutathione‐Sepharose beads. Proteins retained on Sepharose were then subjected to immunoprecipitation with indicated antibodies. H,I) Schematic illustration of USP10 structure (H) and the indicated plasmids were transfected into HEK293T cells for 48 h, then cells were lysed and subjected to immunoprecipitation with Flag magnetic beads or His magnetic beads (I). J) The indicated plasmids were transfected into HEK293T cells for 48 h, then cells were lysed and subjected to immunoprecipitation with Flag magnetic beads. For all panels, data are representative results of three independent experiments.

To illuminate the possible interaction between USP10 and POLR2A, affinity purification and mass spectrometry was employed to interrogate interactome of POLR2A. These data demonstrated that POLR2A correlated with multiple USPs, including USP5, USP7, USP8, and USP10 etc, of which USP10 had the highest score (**Figure** **6A,B**; Table S6 and Figure S7A, Supporting Information). Utilizing the AlphaFold3 algorithm,^[^ ^36^ ^]^ we found that multiple interaction sites between USP10 and POLR2A that were characterized by complementary surface features at the binding interface (Figure S7B, Supporting Information). Immunofluorescence staining showed that USP10 pervasively colocalized with POLR2A in Fadu and SAS cells (Figure 6C). Coimmunoprecipitation (CoIP) experiments validated that POLR2A physically interacted with USP10 in 293T cells and Cal27 cells that transfected with Flag or His tagged plasmids of USP10 and POLR2A (Figure 6D,E). Similarly, endogenous POLR2A was also combined with USP10 in Fadu cells (Figure 6F). Moreover, purified GST‐USP10, rather than the GST control, was able to bind to HIS‐tagged POLR2A under cell‐free conditions (Figure 6G), indicating a direct interaction between USP10 and POLR2A.To further assess which structural domains of USP10 and POLR2A mediate this interaction, we mutated the regions of USP10 that are potentially associated with the interaction with POLR2A (Figure 6H). We found that their interaction was controlled by 100–400 amino acids in the N‐terminal fragment of USP10 (Figure 6I), as no interaction was observed after the ablation of this domain. In addition, we also validated that K1268R, a known ubiquitination modification site of POLR2A,^[^ ^37^ ^]^ was required for the direct interaction with USP10 (Figure 6J).

### 2.6 USP10 Removes K48‐ and K63‐Linked Ubiquitin Chains of POLR2A through its Deubiquitinase Activity

**Figure 7:**
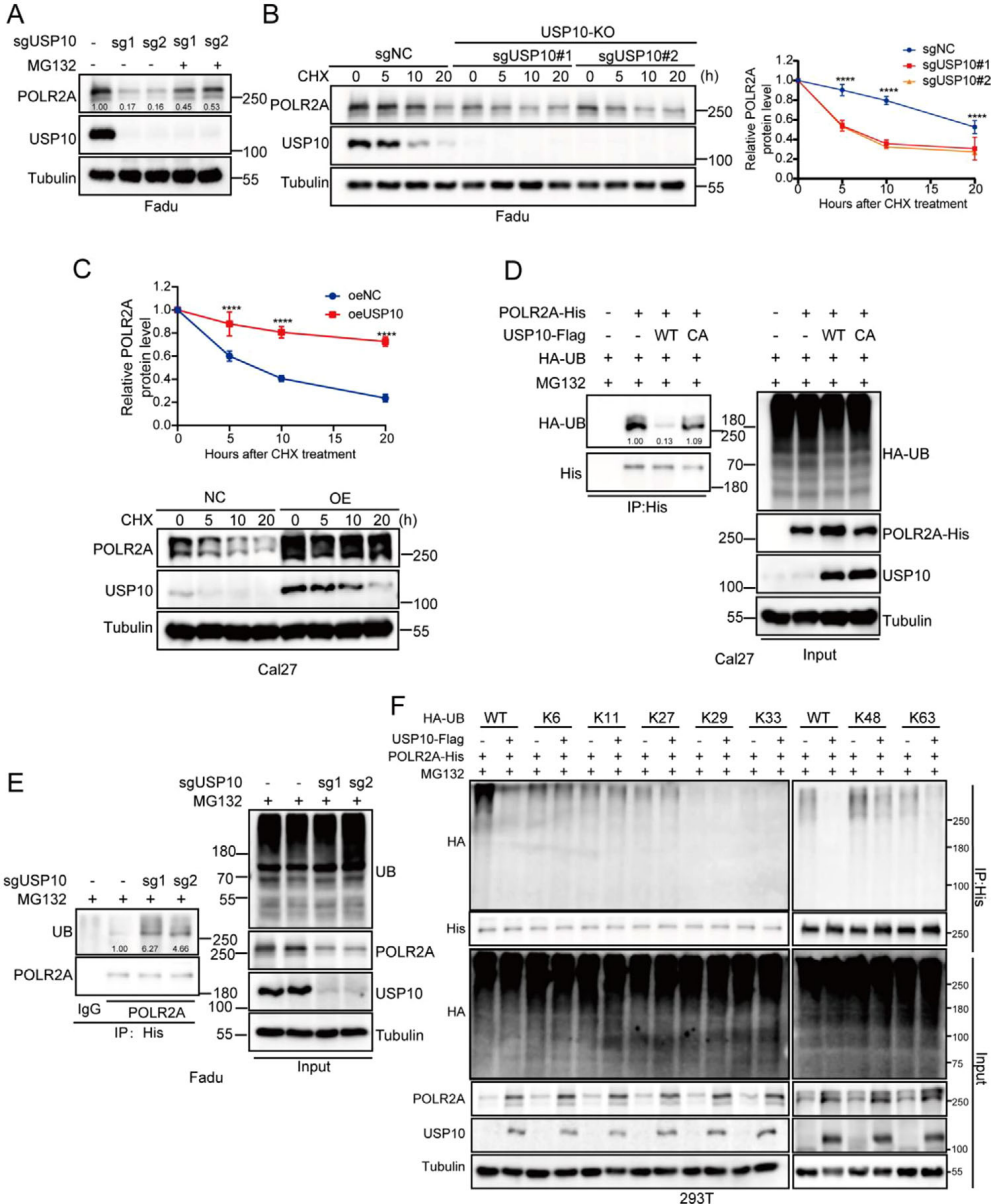
USP10 removes K48‐ and K63‐linked ubiquitin chains of USP10 through its deubiquitinase activity. A,B) USP10 depleted Fadu cells were treated with MG132 (10 µm) for 12 h (A), or treated with cycloheximide (CHX) (50 µg mL^−1^) (B); left, immunoblot bands; right, quantitative relative POLR2A protein level for stated divergent time points. USP10 and POLR2A proteins were detected by immunoblot. C) Cal27 cells stably expressing Flag‐vector or Flag‐USP10 were treated with CHX (50 µg mL^−1^) at different time points. POLR2A protein level was detected by immunoblot (upper, immunoblot bands; lower, quantitative relative level of POLR2A protein). D) Cal27 cells were co‐transfected with His‐POLR2A, HA‐Ub, and Flag‐USP10 WT or Flag‐USP10 C424A; then cell lysates were subjected to IP with magnetic beads, followed by immunoblot with indicated antibodies. Cells were treated with 10 µm MG132 for 10 h. E) USP10 depleted Fadu cells were transfected with the indicated plasmids and treated with MG132 (10 µm) for 12 h before harvesting, and cell lysates subjected to IP with His magnetic beads, followed by immunoblot with indicated antibodies. F) HEK293T cells were transfected with the indicated plasmids and treated with MG132 (10 µm) for 12 h before harvesting, followed by immunoblot with indicated antibodies. All data are representative of at least three independent experiments. Data are presented as mean ± SD, *n* = 3. *p* value was determined by two‐way ANOVA; ns, not significant (*p* > 0.05); * *p* < 0.05; ** *p* < 0.01; *** *p* < 0.001; **** *p* < 0.0001.

We did not observe alterations in the expression of *POLR2A* mRNA in USP10‐depleted HNSCC cells (Figure 5C). This data suggests that POLR2A is possibly to be ubiquitinated by USP10. To confirm this hypothesis, we found that the addition of a proteasomal inhibitor MG132 restored POLR2A protein expression in USP10‐depleted cells (**Figure** **7**A; Figure S7C, Supporting Information). Next, a protein synthesis inhibitor cycloheximide (CHX) was used to confirm the regulatory effect of USP10 on POLR2A stability, and we observed that the degradation of POLR2A was positively correlated to the reduction of USP10 expression in both USP10 depleted and overexpressed HNSCC cells (Figure 7B,C; Figure S7D,E, Supporting Information). Enforced expression of USP10‐WT, but not USP10‐C424A (USP10 inactive mutation), resulted in a prominent increase in the stability of ectopically expressed POLR2A protein in HNSCC cells (Figure 7D). In USP10‐depleted Fadu cells, we noted that a significantly elevated ubiquitination of POLR2A when compared to that of control cells (Figure 7E; Figure S7F, Supporting Information). Moreover, a significantly reduced ubiquitination of POLR2A in USP10 overexpressing Cal27 cells, but not in cells overexpressing mutant C424A‐USP10 (Figure 7D). To further confirm the ubiquitin linkage of POLR2A regulated by USP10, we performed a deubiquitylation assay of POLR2A by co‐transfection with a series of ubiquitin mutants. Interestingly, USP10 specifically removed K48‐ and K63‐ linked ubiquitin chains of POLR2A (Figure 7F).

To prove clinical relevance of the USP10‐POLR2A‐SLC7A11 axis, we finally assessed POLR2A and SLC7A11 proteins in HNSCC samples via immunohistochemistry (Figure S8A,B, Supporting Information). Indeed, a positive correlation between USP10 and POLR2A, and similar positive correlation between USP10 and SLC7A11 were identified (Figure S8C, Supporting Information). Moreover, as shown in Table S7 (Supporting Information), POLR2A expression correlated with alcohol consumption, clinical stage, and metastasis (All *p* < 0.05); SLC7A11 expression was associated with T classification, clinical stage, and metastasis (All *p* < 0.05). Survival analysis results revealed that high expression of POLR2A (not SLC7A11) predicted a worse prognosis in HNSCC patients (Figure S8D,E, Supporting Information). Taking into consideration the expression of POLR2A and SLC7A11, the multivariate Cox regression analysis still confirmed that USP10 expression and metastasis were independent prognostic parameters in patients with HNSCC (Table S8, Supporting Information). Together, these results provide solid evidence to support a novel USP10‐POLR2A‐SLC7A11 axis as a key signaling pathway to regulate cancer ferroptosis.

## 3 Discussion

Cancer cells that are resistant to conventional agents of chemotherapy show superior sensitivity to ferroptotic inducers, suggesting that promoting ferroptosis in these cancer cell populations is a rationally promising strategy for cancer target therapy.^[^ ^5^, ^6^ ^]^ DUBs remove ubiquitins from their substrates and change the abundance of target proteins, therefore, they play a key role in the maintenance of protein homeostasis. DUBs have definite catalytic clefts in structure, which are intrinsically appealing sites for drug targeting.^[^ ^8^, ^9^ ^]^ Thus, deciphering the regulatory mechanisms of DUBs‐mediated ferroptosis in the cancer scenario will accelerate the discovery of novel agents in this field.

DUBs regulate ferroptosis in diverse noncancerous diseases, including intervertebral disc degeneration,^[^ ^16^ ^]^ chronic obstructive pulmonary disease,^[^ ^19^ ^]^ ischemia/reperfusion‐induced renal injury,^[^ ^13^ ^]^ myocardial injury,^[^ ^14^ ^]^ spinal cord injury,^[^ ^12^, ^18^ ^]^ etc. In these studies, DUBs deubiquitinate and delay the degradation of target proteins, which in turn disturbs the balance of ferroptosis promoting and/or defense system.^[^ ^12^, ^13^, ^14^, ^16^, ^18^, ^19^ ^]^ In the field of cancer investigations, USP5 interacts with Lymphoid‐specific helicase (LSH), USP8 binds to β‐catenin, USP11 interacts with LSH, enhances the protein stability through deubiquitination, which consequently promotes cancer development by suppressing ferroptosis.^[^ ^11^ ^]^ Collectively, instead of direct modification of key ferroptotic regulators, majority of available studies indicate that DUBs mainly function on the upstreaming proteins; however, OTUB1 can directly enhance the stability of SLC7A11, a key ferroptosis defense protein.^[^ ^38^ ^]^

In this study, USP10 was determined to be a potential ferroptosis regulator, which was in agreement with the genome‐wide CRISPR screen system reported by Zou Y et al.^[^ ^39^ ^]^ Gene gain and loss of functional experiments confirmed that USP10 suppressed ferroptosis via a SLC7A11‐dependent way in HNSCC, not through other pathways (DHODH and FSP1). Conceivably, gene knockout or antagonist targeting USP10 sensitized HNSCC cells to ferroptosis both in vitro and in vivo. In agreement with previous studies, USP10 acts as an oncoprotein in hepatocellular carcinoma,^[^ ^20^ ^]^ esophageal squamous cell carcinoma,^[^ ^21^ ^]^ glioblastoma,^[^ ^23^ ^]^ and acute myeloid leukemia^[^ ^26^ ^]^ through the stabilization of YAP, ANLN, RUNX1, and FLT3. In contrast, USP10 also exerts a tumor suppressive role in lung cancer and in colorectal cancer via modulating the ubiquitin of KLF4 and ZEB1.^[^ ^22^, ^25^ ^]^ Based on these findings, we can conclude that the effects of USP10 on cancer malignant behaviors are context‐specific, and always depends on the key protein substrates that regulated by USP10‐mediated deubiquitination.

To date, the links and molecular mechanisms between USP10 and ferroptosis have been scarcely investigated. Here, we determine that USP10 represses ferroptosis via increasing SLC7A11 expression at both transcriptional and protein levels. However, no mutual interaction between USP10 and SLC7A11 was identified, which excluded the possibility that USP10 might directly increase the expression of SLC7A11 via its deubiquitination, raising the hypothesis that a potential intermediate regulator may be involved in the USP10‐mediated transcriptional activation of *SLC7A11*. To determine the intermediate protein, we simultaneously analyzed the potential interaction proteins of USP10, and proteins that may bind to the promoter regions of *SLC7A11*. Ultimately, POLR2A as a candidate was identified.

POLR2A encodes the largest subunit of RNA polymerase II complex in eukaryotes and drives the transcription of mRNAs.^[^ ^40^, ^41^, ^42^ ^]^ Inhibition of POLR2A induces cell senescence,^[^ ^43^ ^]^ and reduces the tumor growth in triple negative breast cancer,^[^ ^41^ ^]^ colorectal cancer,^[^ ^42^ ^]^ and gastric cancer,^[^ ^44^ ^]^ suggesting that POLR2A is essential for the survival of cancer cells. It has been reported that p53 inhibits cystine uptake and sensitizes cells to ferroptosis by repressing expression of SLC7A11.^[^ ^45^ ^]^ In contrast, as a neighboring gene of *TP53* that co‐localize in the Chr17p region, POLR2A functions in a totally opposite way to transcriptionally activate *SLC7A11*, hence repressing ferroptosis of cancer cells.

Several lines of evidence have been applied in our study to support the notion that USP10 is a true deubiquitinase for POLR2A. Then, Co‐IP, immunofluorescence and GST‐pull down experiments were used to determine their mutual interaction between USP10 and POLR2A. Forced expression of USP10 dramatically elevated the protein level of POLR2A and removed its ubiquitination level, whereas USP10 depletion promoted the protein degradation of POLR2A. Collectively, these data validate that POLR2A is deubiquinated by USP10 via its K48‐ and K63‐linked ubiquitin chains. Combined with the previous report demonstrates that E3 ubiquitin ligase RBX1 is a direct interactor of POLR2A, which promotes the POLR2A‐dependent RNA transcription via a K63‐linked poly‐ubiquitination.^[^ ^46^ ^]^ All together, these data indicate that POLR2A stability is fine‐tuned by the ubiquitin proteasome pathway via the E3 ligases and DUBs system.

It should be noted that our study also validates that patients with USP10 or POLR2A overexpression have a worse survival prognosis. POLR2A mutation was a poor prognostic marker associated with the recurrence of cerebellopontine angle (CPA) meningioma^[^ ^47^ ^]^; our study verified that high expression of POLR2A protein predicts a short survival time in HNSCC patients. Regarding to USP10, its overexpression is positively associated with a poor prognosis in glioblastoma,^[^ ^23^ ^]^ esophageal squamous cell carcinoma,^[^ ^21^ ^]^ pancreatic cancer,^[^ ^24^ ^]^ et al., while its overexpression has negative clinical relevance in lung cancer,^[^ ^22^ ^]^ indicating a cancer‐specific links in the prediction of cancer prognosis. Our data clearly confirm that USP10 expression is positively associated with lymph node metastasis, which supports that USP10's association with outcome may partially reflect its dual roles in both tumor cellular biology and metastasis. At the molecular level, USP10 and POLR2A are mechanistically linked. This close link precludes a concurrent inclusion of these two proteins in prognostic models. The dominance of only USP10 in multivariate Cox analysis reflects its broader biological relevance beyond POLR2A regulation. Except for its clinical value, we confirm that targeting USP10 (gene depletion and antagonist) inhibits the growth of HNSCC in vitro and in vivo, identification of USP10 as suppressor of ferroptosis could have potential clinical translational implications for cancer target therapy, with the consideration that inhibitors of DUBs have already been approved by the FDA or widely researched in the clinic.

While this study provides solid evidence to support the function of USP10‐POLR2A‐SLC7A11 axis in cancer ferroptosis, it has limitations. Firstly, cancer ferroptosis is always associated with responsiveness to chemo‐ and/or radio‐therapy, the connections between this axis and treatment responsiveness are not clarified in this study. Hence the role of this axis still needs to be validated across different HNSCC subtypes (e.g., chemotherapy responsive or nonresponsive; HPV positive or negative) and diverse cancer types in future, as to whether this signaling axis represents a common mechanism in cancer ferroptosis. Secondly, given that inducing ferroptosis is a promising strategy to instigate immunotherapy, it is intriguing to verify whether USP10 participates in modulating HNSCC immune microenvironment. In this respect, genetically engineered mouse models that develop de novo cancers in natural immune‐proficient microenvironment should be used. Third, the clinical translational potential of targeting this axis for therapy is required to be investigated in preclinical models, such as cancer organoids. Taken together, our data reveal that USP10 is a ferroptosis suppressor with therapeutic potential and clinical value as prognostic factors.

## 4 Experimental Section

### HNSCC Datasets Acquisition and Data Processing

The ferroptosis suppressor genes were first obtained from the ferrdb V2 database (http://www.zhounan.org/ferrdb/current/). The TCGA‐HNSCC gene expression RNAseq Fragments Per Kilobase per Million (FPKM) data and patient survival data (*n* = 501) were then downloaded and collated from the TCGA website (https://portal.gdc.cancer.gov/). Ferroptosis suppressor index (FSI) was calculated by the ssGSEA in the R language “GSVA package.”^[^ ^48^ ^]^ The correlation between 78 ubiquitination enzyme genes and FSI was calculated (Table S1, Supporting Information). Additionally, RNA microarray data and survival information in another 2 HNSCC cohorts (GSE42743, *n* = 103; GSE41613, *n* = 97) were acquired from the Gene Expression Omnibus (GEO) database (https://www.ncbi.nlm.nih.gov/geo/). Counts were normalized via quantile normalization (limma package), and then batch effects between the merged datasets were corrected using the ComBat algorithm (sva package) according to the method described previously.^[^ ^49^ ^]^ The combined GEO dataset served as an independent validation cohort for survival analysis. Detailed information on treatment and clinical parameters of HNSCC patients in public datasets of TCGA, GSE42743, and GSE41613 is provided in Table S2 (Supporting Information).

### Human Clinical Specimens

According to the previous studies,^[^ ^50^, ^51^, ^52^ ^]^ specimens and clinical information from 167 patients with HNSCC (ranged from January 2000 to October 2005) were collected in the department. All enrolled patients were included according to the following criteria: no previous history of radio‐ or chemotherapy, and primary HNSCC without other malignancies. The study was conducted in accordance with the Declaration of Helsinki and approved by the Research Ethics Committee of Xiangya Hospital, Central South University. Written informed consents were obtained from all patients. Following‐up materials after surgery were obtained from 164 (98.2%) patients, and three patients lost to follow‐up because of telephone number changes or home moving.

### Immunohistochemistry (IHC)

As previously reported,^[^ ^50^, ^51^, ^52^ ^]^ formalin‐fixed, paraffin‐embedded (FFPE) sections from patient or mouse were deparaffinized, dehydrated, and subjected to antigen retrieval in 10 mm citrate buffer (pH 6.0) using a microwave oven. After 15 min of antigen retrieval, endogenous peroxidase activity was quenched with 3% hydrogen peroxide for 10 min at room temperature. Thereafter, samples were incubated overnight at 4 °C with anti‐USP10 (Proteintech, 67917‐1‐Ig), anti‐POLR2A (Invitrogen, MA5‐42551), SLC7A11 (Invitrogen, MA5‐44963). Immunostaining was performed using a two‐step protocol and a PV‐9000 Polymer Detection System (ZSGB‐BIO, Beijing, China), according to the manufacturer's instructions. Subsequently, the slides were incubated with 0.1 mL DAB for 2–5 min, and the cell nucleus was counterstained with Mayer's hematoxylin.

### IHC Staining Evaluation

All slides were evaluated and scored by 2 board‐certificated pathologists in the hospital (Xueping Feng and Desheng Xiao), who were blinded to clinical information. If a disagreement occurred, the slides were re‐examined to achieve a final consensus. The staining intensity was scored as follows: 0 (no staining), 1 (slightly brown), 2 (moderately brown), and 3 (dark brown). According to the percentages of positive staining areas in relation to the entire carcinoma‐involved area, the extent of staining was scored as 0 (0%), 1 (1–25%), 2 (26–50%), 3 (51–75%), and 4 (76–100%). Finally, a staining score (SI) was multiplied by the above two scores. Based on the SI values, protein expression level was categorized as low expression (SI = 0–5) and high expression (SI = 6–12), in which the cutoff value for high and low expression was determined by measuring heterogeneity with statistical analysis of log‐rank test regarding the overall survival.^[^ ^52^ ^]^

### Cell Culture and Culture Conditions

The oral mucosal precancerous lesions cell (DOK), HNSCC cell lines (Fadu, Detroit562, Tca8113, Tu212 and Cal27) and the human embryonic kidney 293T were purchased from the American Type Culture Collection (ATCC) (Manassas, VA, USA) or the Cell Bank of Type Culture Collection of Chinese Academy of Sciences (Shanghai, China). SAS was obtained from the Japanese Collection of Research Bioresources Cell Bank (Tokyo, Japan). Tu686 was generously provided by Dr. Zhuo Chen (Winship Cancer Institute, Emory University School of Medicine, Atlanta, GA, USA).^[^ ^53^ ^]^

DOK, Tca8113, and Tu212 were cultured in RPMI 1640 medium (Gibco, Thermo Fisher Scientific, 11875119, China). Tu686 was cultured in Dulbecco's modified Eagle's medium DMEM/F12 medium (Gibco, 11320033), and the remaining cells were cultured in DMEM medium (Gibco, 11965092). Cell medium was supplemented with 10% fetal bovine serum (FBS) (Corning, 35‐010‐CV, USA) plus 1% penicillin and streptomycin (Gibco, 1514022; Massachusetts, USA). All cells were incubated at 37 °C under an atmosphere of 5% CO_2_. All cell lines were routinely excluded for mycoplasma contamination, and cells in the exponential phase were used in the following experiments.

### Antibodies and Chemicals

Antibody information was listed as follows: USP10 (Proteintech, 19374‐1‐AP and 67917‐1‐Ig; Shanghai, China; CST, #8501), SLC7A11 (CST, #12691), GPX4 (Proteintech, 67763‐1‐Ig), FSP1 (Proteintech, 20886‐1‐AP), DHODH (Proteintech, 14877‐1‐AP), POLR2A (Proteintech, 20655‐1‐AP and CST, #14958) and GST‐tag (CST, #2622). Ubiquitin (CST, #3936), GAPDH (CST, #5174), Tubulin (CST, #5568), Actin (Proteintech, 81115‐1‐RR), Flag‐tag (CST, #14793), His‐tag (CST, #12698), HA‐tag (CST, #3724), Ki67 (Proteintech, 27309‐1‐AP), 4 Hydroxynonenal (4‐HNE) (Abcam, ab46545), HRP‐labeled secondary antibody (CST, #7074S, #7076S), Mouse Anti‐rabbit IgG mAb (CST, #3678).

### Chemical Compounds

Erastin (Selleck, S7242), RSL3 (Selleck, S8155), Imidazole ketone Erastin (IKE) (Selleck, S8877), Ferrostatin‐1 (Fer‐1) (Selleck, S7243), Z‐VAD‐FMK (Selleck, S7023) and 3‐Methyladenine (3‐MA) (Selleck, S2767).

### Colony Formation and Cell Viability Assay

For colony formation assay, Fadu and SAS cells were incubated with Erastin (10 µm), Erastin (10 µm) and Fer‐1 (1 µm), Erastin (10 µm) and Z‐VAD‐FMK (Z‐VAD, 20 um), or Erastin (10 µm) and 3‐methylademine (3‐MA, 5 mm) for 12 h in 6‐well or 12‐well plates. Subsequently, the normal complete medium was replaced and cultured for another 1–2 weeks until the formation of colonies. Cell colonies were fixed with methanol, stained with crystal violet, and finally quantified by measuring the absorbance of crystal violet at 592 nm.

Cell viability was measured by cell counting kit‐8 (CCK‐8) assay (Biosharp, BS350A; Beijing, China). Briefly, cells were seeded into a 96‐well plate and incubated with indicated treatment. Subsequently, 100 µl fresh culture medium containing 10 µl of CCK‐8 solution was added to the cells and incubated for 1 h (37 °C, 5% CO_2_). Then, absorbance was measured at 450 nm using a spectrophotometer. The collected values were normalized to the blank well, and relative cell viability was normalized to the respective DMSO treatment well.

### Cell Death Assay

Following different stimuli, cells were harvested using trypsin, washed twice with PBS, and stained with 5 µg mL^−1^ propidium iodide (PI) (BD Biosciences, 556463). Cells were imaged on a BD Biosciences LSRFortessa, and were analyzed with the FlowJo software (v10.4.0).

### Lipid ROS Measurement

Cells in 12‐well plates (4 × 10^5^ cells per well) were treated with DMSO, Erastin, RLS3, or Fer‐1 for 10 h. Then BODIPY‐581/591 C11 (Thermo, D3861) was added to the medium at a concentration of 10 µm, and incubated for 30 min at 37 °C, 5% CO_2_. Subsequently, cells were gathered using trypsin, washed twice with PBS, and resuspended in 200 µl PBS. Lipid ROS levels were finally detected using a BD Biosciences LSRFortessa, and data were processed with the FlowJo software (v10.4.0).

### Ferrous Ion Content Assay

Ferrous ion changes were assessed using a ferrous ion content assay kit (Solarbio, BC5415) according to the manufacturer's instructions.

### Mitochondrial Membrane Potential Measurement

Mitochondrial potential changes were assessed using a mitochondrial membrane potential measurement kit containing JC‐1 (Beyotime, C2006) according to the manufacturer's instructions. After incubation with JC‐1 staining working solution at 37 °C for 20 min, cells were washed twice with JC‐1 staining buffer (1X). The medium was then replaced with normal medium, and cells were imaged using a fluorescence microscope.

### Plasmid Construction and Transfection

CRISPR‐mediated knockout plasmids containing guide RNAs targeting USP10 were generated in LentiCRISPRV2 (Addgene, #52961). Flag‐USP10‐WT, Flag‐USP10‐1 (1‐100), Flag‐USP10‐2 (100‐400) and Flag‐USP10‐3 (400‐782) were cloned into the plasmid vector pLVX‐Puro. GST‐tagged USP10 was cloned into the pGEX‐4T‐1 vector. POLR2A‐WT‐HIS (Addgene, #139404), POLR2A‐K1268R‐HIS (Addgene, #139405), Flag‐HA‐USP10_C424A (Addgene, #127103) and Flag‐HA‐USP10 (Addgene, #127102) were purchased from Addgene (MA, USA). SLC7A11‐Promotor (5‐174) were subcloned into the EcoRI and NotI sites of PGL3‐basic plasmids from Tsingke Biotechnology Co., Ltd (Beijing, China), and shRNA‐SLC7A11 was purchased from Tsingke Biotechnology Co., Ltd, Beijing, China.

The sgRNA sequence of Human USP10 targets the Exon 4, sgRNA1: 5’‐GCCTGGGTACTGGCAGTCGATGG‐3’; sgRNA2: 5’‐TCCATCGACTGCCAGTACCCAGG‐3’. Human shSLC7A11 sequence: 5’‐ATAATAAAGAGATAATACG‐3’. Transient silencing of POLR2A was performed using a riboFECT CP Transfection Kit (Ribobio, C10511‐05; Guangzhou, China). The following target sequences were used in the study: control siRNA, 5′‐UUCUCCGAACGUGUCACGU‐3′ and 5′‐ACGUGACACGUUCGGAGAA‐3′; POLR2A‐siRNA‐1, 5’‐CCAACAUGCUGACAGAUAU‐3’ and 5’‐AUAUCUGUCAGCAUGUUGG‐3’; POLR2A‐siRNA‐2, 5’‐CCAAGAAGCGGCUCACACA‐3’ and 5’‐UGUGUGAGCCGCUUCUUGG‐3’.

Plasmids were extracted using the E.Z.N.A.^®^ Endo‐Free Plasmid Mini Kit II (Omega Bio‐Tek, D6950; Guangzhou, China). All constructs were confirmed by DNA sequencing. Virus packaging was performed using the Lenti‐Pac™ HIV lentivirus packaging kit (GeneCopoeia, LT002; MD, USA).

### GSH/GSSG Assay

The ratio of GSH/GSSG was measured according to the manufacturer's instructions (Beyotime Biotechnology, S0053, Shanghai, China).

### Transmission Electron Microscopy

The ultrastructure of mitochondria was observed by a transmission electron microscopy (TEM). Following the diverse treatment, indicated cells were fixed with 2.5% glutaraldehyde in 0.1 M PBS (pH 7.4) for 2.5 h at 4 °C, triply washed with 0.1 M PBS and further fixed by 1% OsO4 for 2 h at 4 °C. Then, cell samples were dehydrated through an ethanol gradient and embedded in Spurr's resin. Subsequently, ultrathin sections were stained with uranyl acetate or lead citrate, ultimately followed by the observation with JEOL 1200EX transmission electron microscopy (JEOL Ltd. Japan). Images were captured for recording and analysis.

### Confocal Microscopy and Immunofluorescence

Indicated cells were stained with 2 µm Rho123 (J&K Scientific, 62669‐70‐9, Beijing, China) at 37 °C and 5% CO_2_ for 2 h to allow mitochondria to be colored. After twice rinsing with 1 mL medium containing 10% FBS, cells were imaged by structured illumination microscopy (SIM; LSM 980 with Airyscan 2, Zeiss; Oberkochen, Germany).

For immunofluorescent staining, slides were fixed with 4% paraformaldehyde for 15 min, permeabilized with 0.3% Triton X‐100 for 10 min, blocked with 5% bovine serum albumin (BSA) for 1 h, sequentially incubated with primary antibodies at 4 °C overnight and secondary antibodies conjugated to Alexa Fluor 488 (CST, #4408) or Alexa Fluor 647 (CST, #4414) for 1 h at room temperature. Finally, the slides were mounted using Vectashield Antifade mounting medium with DAPI (Servicebio, G1407, China). Images were captured by a fluorescence confocal microscope (LSM 980 and Airyscan 2, Zeiss; Oberkochen, Germany).

### Western Blotting

As previously described,^[^ ^50^, ^54^ ^]^ total proteins were extracted using RIPA buffer (NCM biotech, WB3100, China) and protease inhibitors (TargetMol, C0001, USA), and their concentration was determined with a BCA protein assay kit (Beyotime, P0012). Subsequently, the samples were separated on 8–12% sodium dodecyl sulfate‐polyacrylamide (Beyotime, P0012A) and transferred onto a polyvinylidene fluoride membrane (Millipore, IPVH00010, Bedford; MA, USA). The membrane was then incubated overnight at 4 °C with the primary antibody, followed by incubation with HRP‐conjugated secondary antibodies (rabbit or mouse) for 1 h at room temperature. GAPDH and Tubulin were utilized as loading controls. Protein bands were visualized using enhanced chemiluminescence reagents, and the images were captured using Azure biosystems (Azure Biosystems Inc., C500, USA).

### RNA Isolation and qRT‐PCR

As previously described,^[^ ^55^ ^]^ total RNA was isolated using Trizol reagent (Thermo Fisher Scientific, 15596026, CA, USA), and its concentration and quality were assessed using a NanoDrop One spectrophotometer (Thermo Fisher Scientific, CA, USA). First‐strand cDNA synthesis was performed using the Surescript™ First‐Strand cDNA Synthesis Kit (GeneCopoeia, QP057), followed by real‐time PCR using the BlazeTaq™ SYBR Green qPCR Master Mix 2.0 (GeneCopoeia, QP034). Relative expression levels compared to the control were calculated using the 2^‐ΔΔCt^ method with GAPDH as a reference gene.^[^ ^56^ ^]^ PCR primers were listed in Table S9 (Supporting Information).

### Dual‐Luciferase Reporter Gene Assay

Transfections were performed using Lipofectamine 3000 (Thermo Fisher Scientific, L3000015) following the manufacturer's protocol. Human *SLC7A11* promoter fragments were synthesized by Tsingke Biotechnology Co., Ltd. Luciferase reporter activity was measured using the Luciferase Assay System (GeneCopoeia, SPDA‐D010).

### Chromatin Immunoprecipitation (ChIP) Assay

The ChIP assay was performed using the EpiQuik™ Chromatin Immunoprecipitation Kit (P‐2002‐1, Epigentek, NY, USA) following the manufacturer's protocol. An anti‐POLR2A antibody (CST, #14958), normal rabbit IgG (CST, #2729), and Anti‐RNA Polymerase II were employed to capture sheared chromatin. Subsequently, immunoprecipitated DNA was purified using the QIAquick PCR Purification Kit (Qiagen, 28104, Dusseldorf, Germany) and quantitated via real‐time PCR. The primers for the assay are listed in Table S9 (Supporting Information).

### Immunoprecipitation (IP) and Mass Spectrometry

Cells were collected and lysed using IP lysis buffer (NCM biotech, P70100, China) and protease inhibitors (TargetMol, C0001) for 30 min on ice at 4 °C. The lysate was centrifuged at 12 000 × g for 15 min to obtain the supernatant. A portion of the lysate was retained for Western blot analysis. The remaining lysates were incubated overnight at 4 °C with gentle shaking and IP reactions were performed using the indicated antibodies and protein G beads (CST, #37478), or BeyoMag™ IDA‐Ni Magnetic Agarose Beads (Beyotime, P2239) or BeyoMag™ Anti‐His Magnetic Beads (Beyotime, P2135) for His‐Tag Protein Purification, and Anti‐FLAG^®^ M2 Affinity Gel (Sigma, A2220) for Flag‐Tag Protein Purification. All subsequent experimental procedures were carried out in accordance with the manufacturer's instructions. Finally, the bound proteins were eluted by boiling or with FLAG Peptide, and the samples were subjected to Western blot analysis or liquid chromatography with tandem mass spectrometry (LC‐MS/MS) analysis.

### In Vitro GST Pull Down Assay

GST‐tagged USP10 proteins were immobilized with GST‐binding resin in GST lysis buffer for 8 h at 4 °C. After washing twice with GST lysis buffer, His‐tagged POLR2A proteins were introduced, and the mixture was rotated at 4 °C for 6 h. The resin was then washed six times with PBST buffer, boiled in SDS loading buffer, and subsequently analyzed by Western blotting.

### RNA Sequencing

RNA extraction, library construction, sequencing, and data analysis was conducted by BGI, Shenzhen, China.

### Xenograft Tumor Model

In this study, male BALB/C nude mice were purchased from Hunan Slac Jingda Laboratory Animal Co., Ltd (Changsha, Hunan, China),. and housed in the Department of Zoology at Central South University. The Department of Zoology had a specific pathogen‐free (SPF)‐grade animal laboratory and a dedicated room for immunodeficient mouse breeding. Animal procedures were reviewed and approved by the Animal Ethics Committee of Central South University (Changsha, Hunan, China).

### Statistical Analysis

The quantitative data are presented as mean ± standard deviation (SD) of at least three independent experiments or biological replicates. All experimental data were analyzed using either SPSS 24.0, GraphPad Prism 8 software or R (v4.0.2). Statistical significance was analyzed using the unpaired, two‐tailed Student's *t*‐test or two‐way ANOVA test. The correlation was analyzed using a Pearson correlation test. Survival curves were generated using the Kaplan–Meier method and compared using the log‐rank test. Cox regression analysis was performed for both univariate and multivariate analyses to determine the independent prognostic value of the variables. Statistical significance was set at *p* < 0.05.

## Conflict of Interest

The authors declare no conflict of interest.

## Author Contributions

Y.L. and Z.F.L. contributed to conceptualization. Y.L., Z.F.L., M.C.H., D.K.Z., and X.Z. were responsible for methodology and software. D.K.Z., Z.F.L., X.Y.W., H.L.Y., W.H.Y., S.H.L., and Y.G. performed validation. D.K.Z., X.Y.W., G.C.Z., Z.N.Y., G.L., and Z.F.L. carried out formal analysis and investigation. Y.L., M.C.H., J.W.H., and X.Z. provided resources. D.K.Z., Z.F.L., X.Y.W., and Y.L. curated the data. Y.L., D.K.Z., and Z.F.L. prepared the original draft. Y.L., J.W.H., M.C.H., and X.Z. reviewed and edited the manuscript. D.K.Z., Z.F.L., and X.Y.W. performed visualization. Y.L., M.C.H., and Z.F.L. supervised the project and managed administration. Y.L., M.C.H., X.Z., and Z.F.L. acquired funding. All authors approved the final version of the manuscript.

## Supporting information



Supporting Information

Supporting Information

## Data Availability

The data that support the findings of this study are available from the corresponding author upon reasonable request.
