# Supplementary material for: USP10 Inhibits Ferroptosis via Deubiquinating POLR2A in Head and Neck Squamous Cell Carcinoma
Source: Adv Sci (Weinh). 2025 Jul 2;12(36):e12271. doi: 10.1002/advs.202412271 (PMC12462914; doi:10.1002/advs.202412271)
Supplement: Supplementary file 1 — Supporting Information [file ADVS-12-e12271-s001.docx]

**Supporting Information 1**

**USP10 Inhibits Ferroptosis via Deubiquinating POLR2A in Head and Neck Squamous Cell Carcinoma**

*Diekuo Zhang ^1, 2, 3^, Xueying Wang ^1, 2, 3^, Shanhong Lu ^1, 2, 3^, Yan Gao ^1, 2, 3^, Gangcai Zhu ^1, 2, 3^, Guo Li ^1, 2, 3^, Zongnan Yu ^1, 2, 3^, Junwei Hou ^1, 4, 6, 7^, Helei Yan ^1, 2, 3^, Wenhui Yuan ^1, 2, 3^, Xin Zhang ^1, 2, 3, 4^, Mien-Chie Hung ^8, 9, #^, Zhifeng Liu ^1, 2, 3, 5, #^, Yong Liu ^1, 2, 3, 4, #^*

**Table of contents**

**Figure S1.** Prognosis analysis of *PSMD7* and *USP10* in HNSCC patients.

**Figure S2.** Stable USP10-depleted HNSCC cells.

**Figure S3.** USP10 ablation promotes HNSCC ferroptosis.

**Figure S4.** USP10 knockout promotes HNSCC ferroptosis and can only be reversed by a ferroptosis inhibitor.

**Figure S5.** USP10 knockout promotes IKE-induced ferroptosis in vivo.

**Figure S6.** USP10 overexpression inhibits HNSCC ferroptosis sensitivity.

**Figure S7.** USP10 Stabilizes POLR2A through its deubiquitinase activity.

**Figure S8.** Clinical significance of POLR2A and SLC7A11 in HNSCC patients.


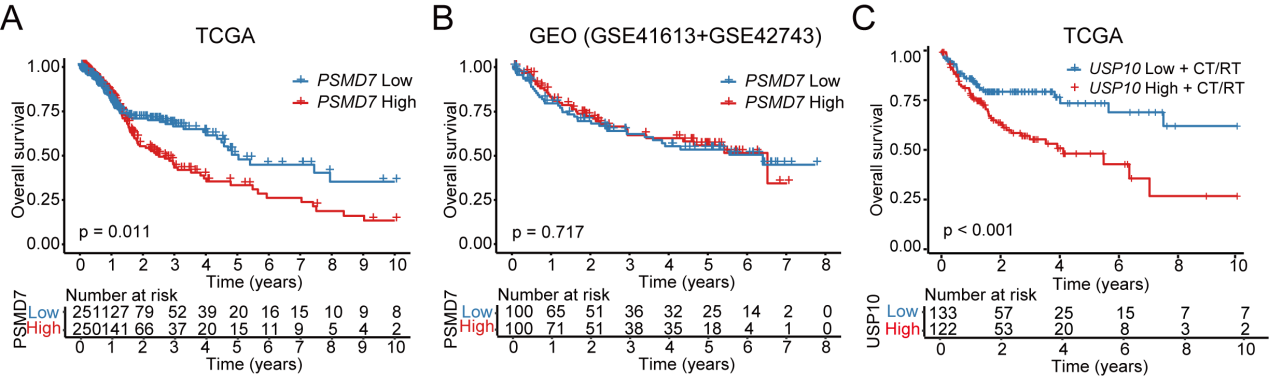


**Figure S1.** Prognosis analysis of *PSMD7* and *USP10* in HNSCC patients.

A-B) Kaplan-Meier curve analysis for correlations between *PSMD7* expression and overall survival of HNSCC patients in TCGA (A, n = 501) and GEO dataset merged by GSE41613 and GSE42743 (B, total n = 200). C) Kaplan-Meier curve analysis for correlations between USP10 expression and overall survival of HNSCC patients in TCGA dataset treated with single radiotherapy and radio- plus chemo-therapy (n = 255). The log-rank test was used to calculate the *p* value.


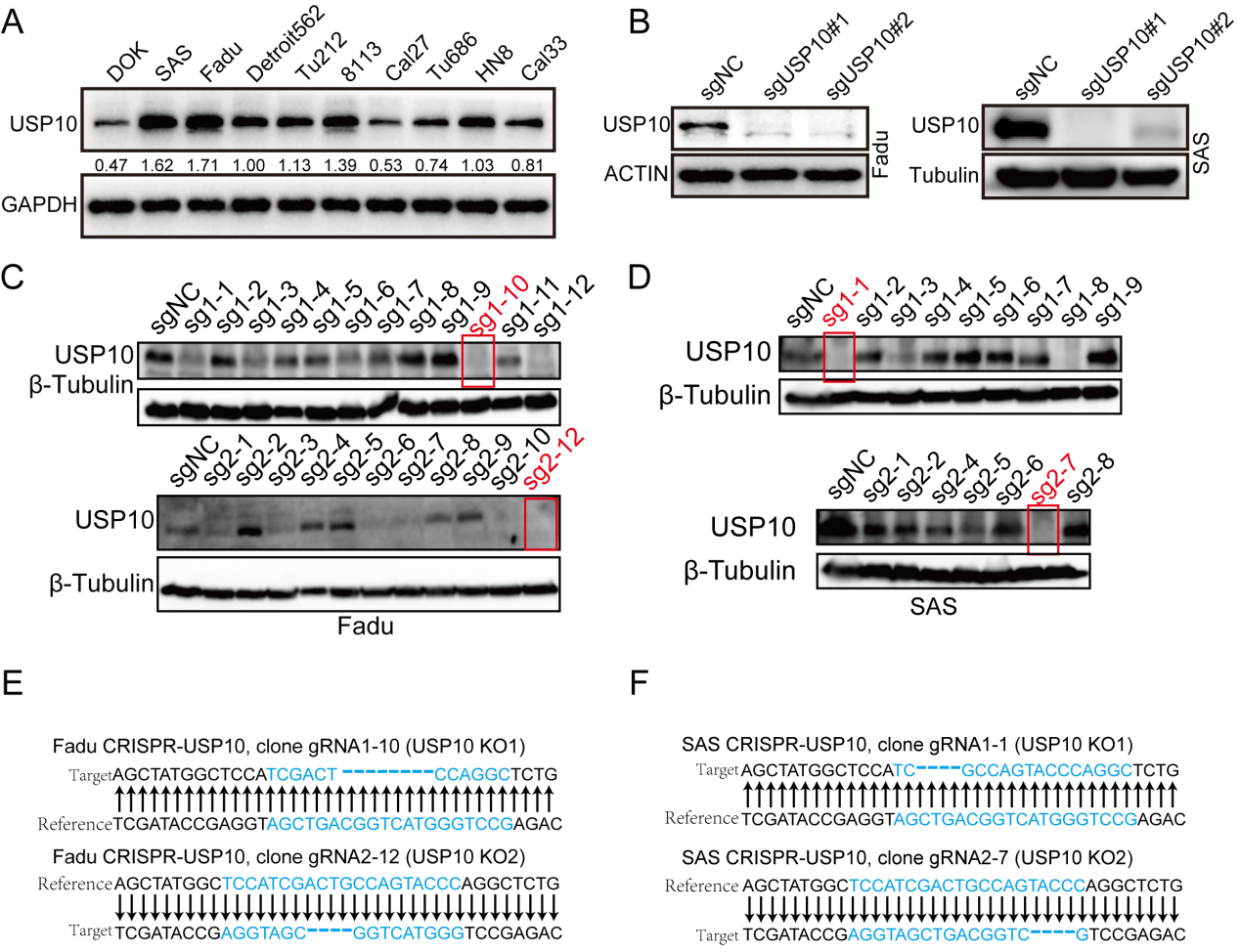


**Figure S2.** Stable USP10-depleted HNSCC cells.

A) USP10 protein level was examined by western blot in indicated precancerous and HNSCC cell lines. B) Fadu (left) and SAS (right) cells were subjected to USP10 depletion with 2 sgRNAs mediated by CRISPR-Cas9 gene knockout system. C and D) Immunoblot of USP10 depletion efficiency in Fadu (C) and SAS (D) clones. E and F) Sequencing results of genomic USP10 exon targeted by sgRNAs (highlighted sequences with color blue) in corresponding Fadu (E) and SAS (F) clones and in alignment with reference sequences.


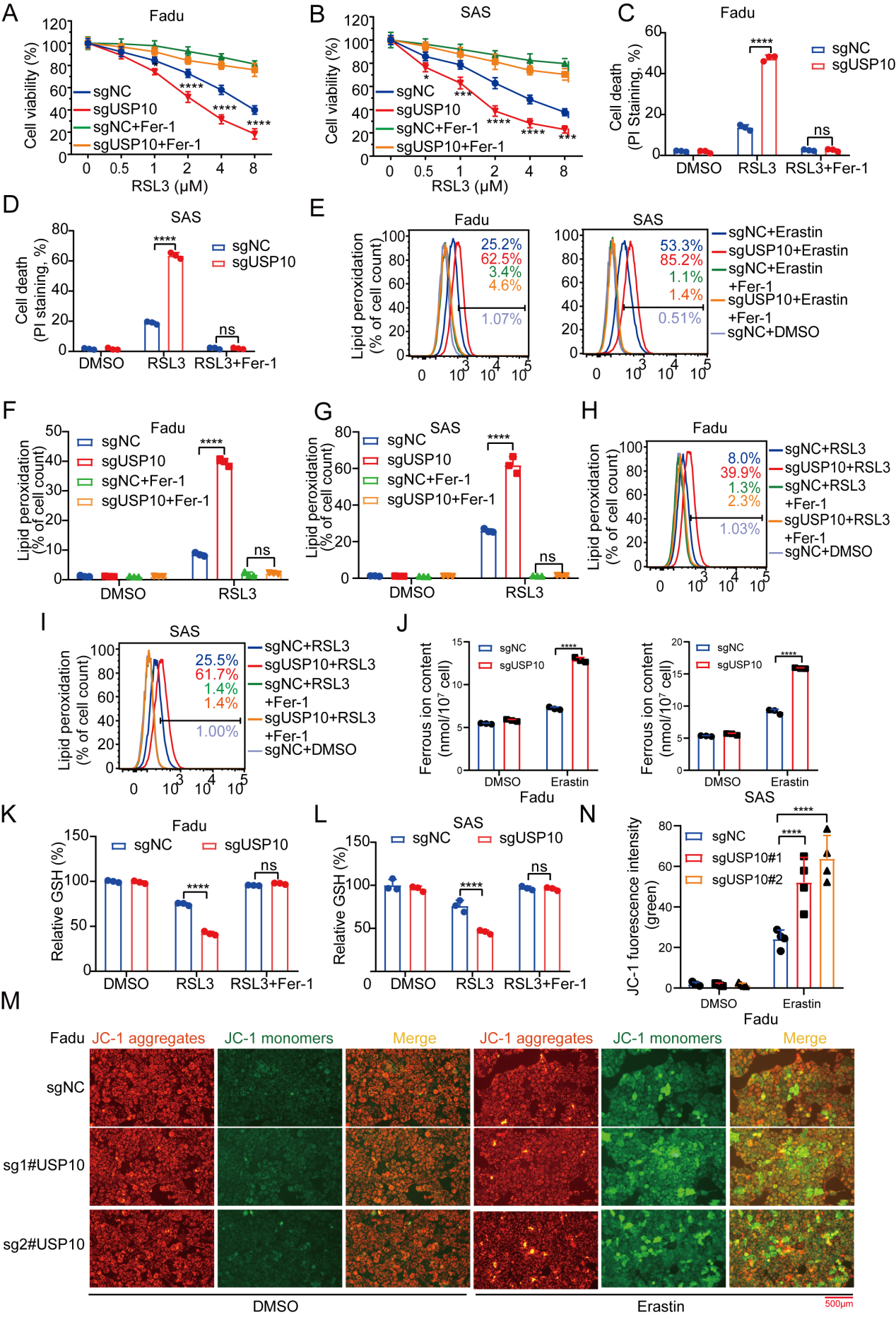


**Figure S3.** USP10 ablation promotes HNSCC ferroptosis.

A-B) Indicated HNSCC cell lines were treated with different concentrations of RSL3 for 48 h, and cell viability was detected by CCK8 assay. C-N) Fadu and SAS cells were treated with 10 μM Erastin or 2 μM RSL3 with or without the addition of 1 μM Fer-1. Then cell death (C, D) was analyzed by PI staining, lipid peroxidation levels (E-I) were detected by BODIPY-C11 probes, labile iron levels (J) were quantified by Ferrous Ion Assay Kit, GSH levels (K, L) were detected with GSH and GSSG assay kits. M, N) Cells were stained for JC-1 to determine the percentage of JC-1 red-negative and green-positive cells (n = 4). Scale bar, 500 μM. All data are representative of at least three independent experiments. Data are presented as mean  ±  SD, n  =  3. *p* value was determined by 2-way ANOVA; ns, not significant (*p* > 0.05); *, *p* < 0.05; **, *p* < 0.01; ***, *p* < 0.001; ****, *p* < 0.0001.


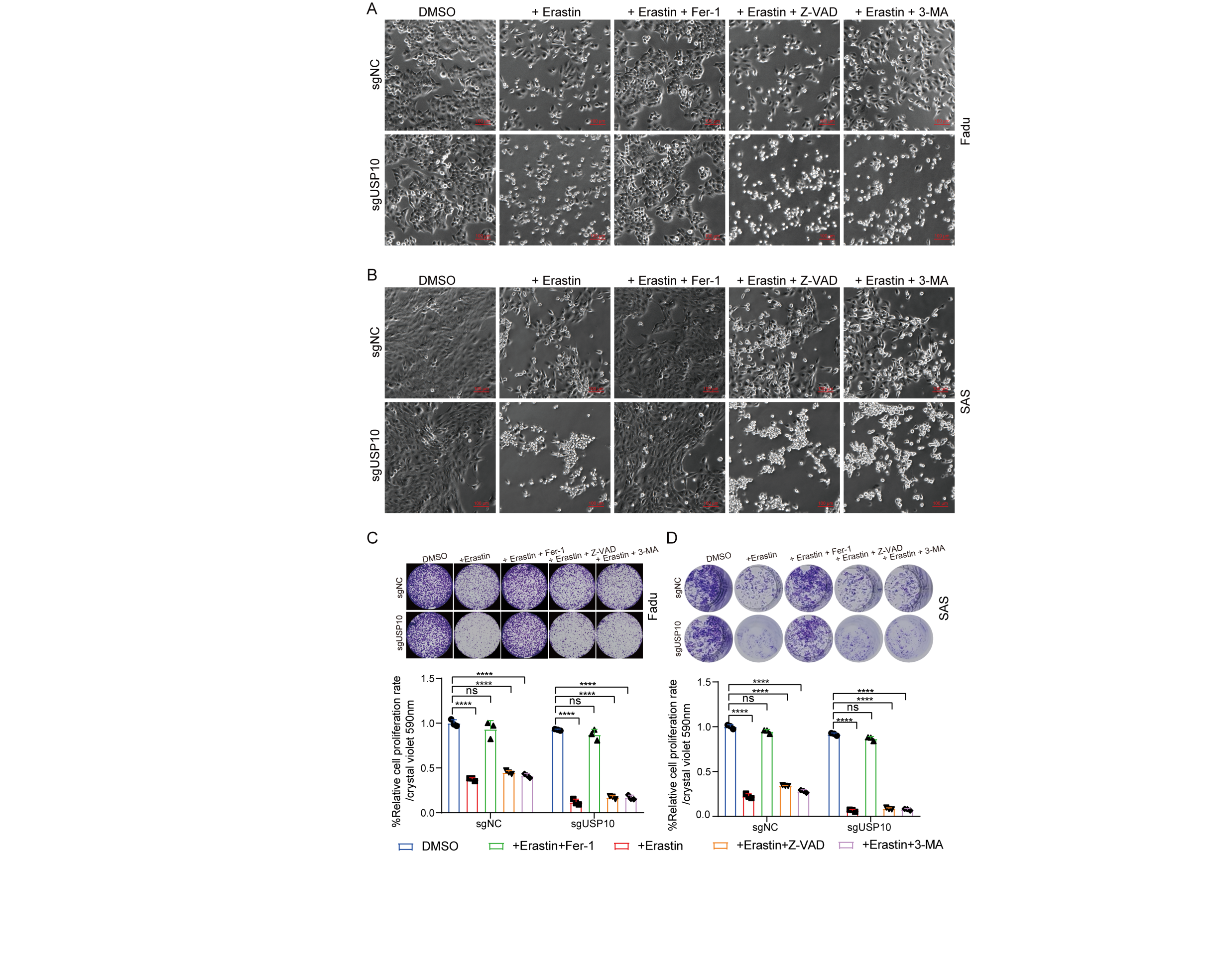


**Figure S4.** USP10 knockout promotes HNSCC ferroptosis and can only be reversed by a ferroptosis inhibitor.

A-D) Representative phase-contrast images and clonogenic analysis of Fadu (A, C) and SAS (B, D) cells were treated with Erastin, Erastin and Fer-1, Erastin and Z-VAD-FMK (Z-VAD), or Erastin and 3-methylademine (3-MA). Scale bar, 100 μM. All data are representative of at least three independent experiments. Data are presented as mean  ±  SD, n  =  3. *p* value was determined by 2-way ANOVA; ns, not significant (*p* > 0.05); ****, *p* < 0.0001.

**
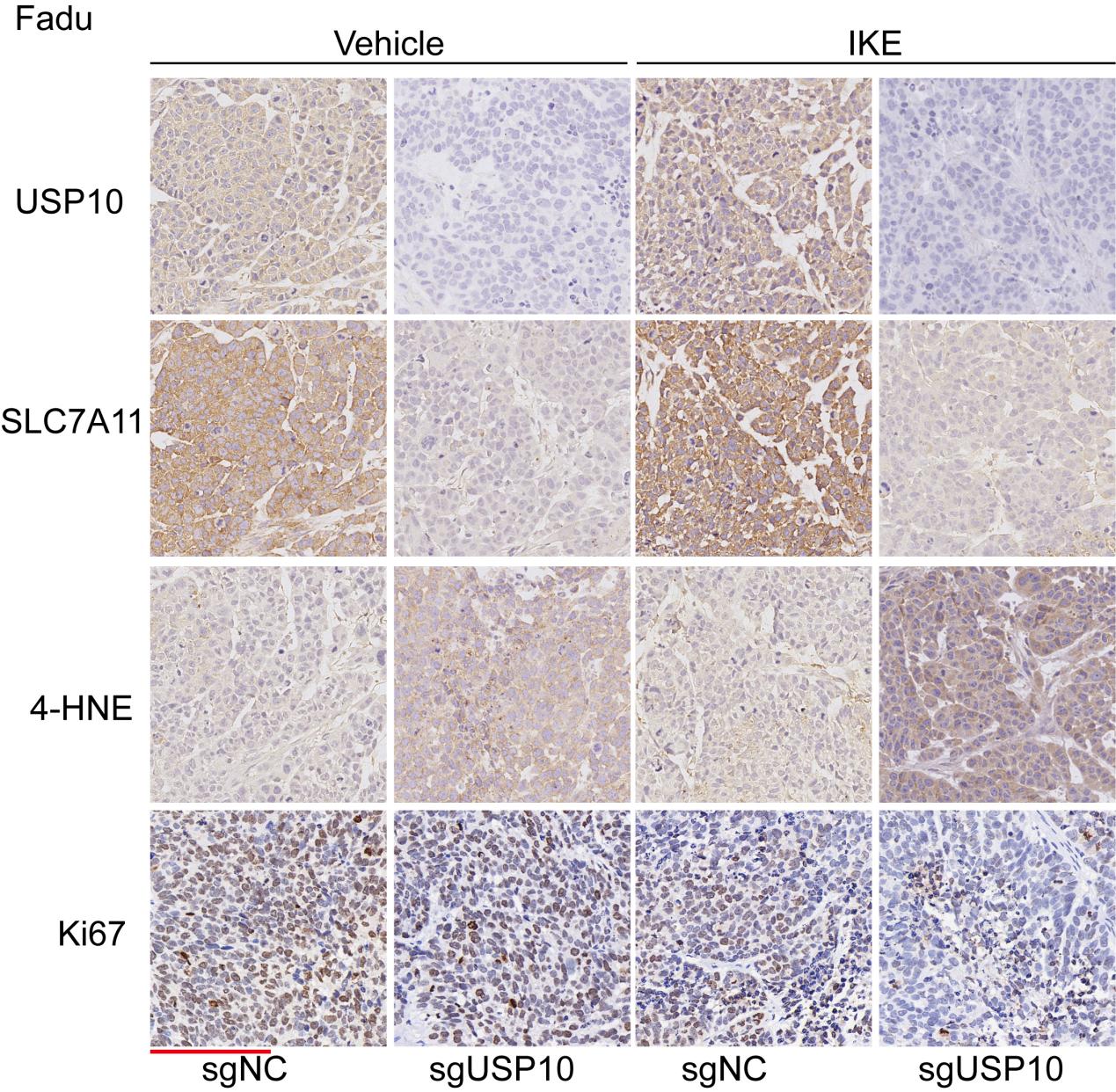
**

**Figure S5.** USP10 knockout promotes IKE-induced ferroptosis in vivo.

Representative immunohistochemical images of USP10, SLC7A11, Ki67 and 4-HNE in the indicated mice tumor sections. Scale bar, 100 μm.


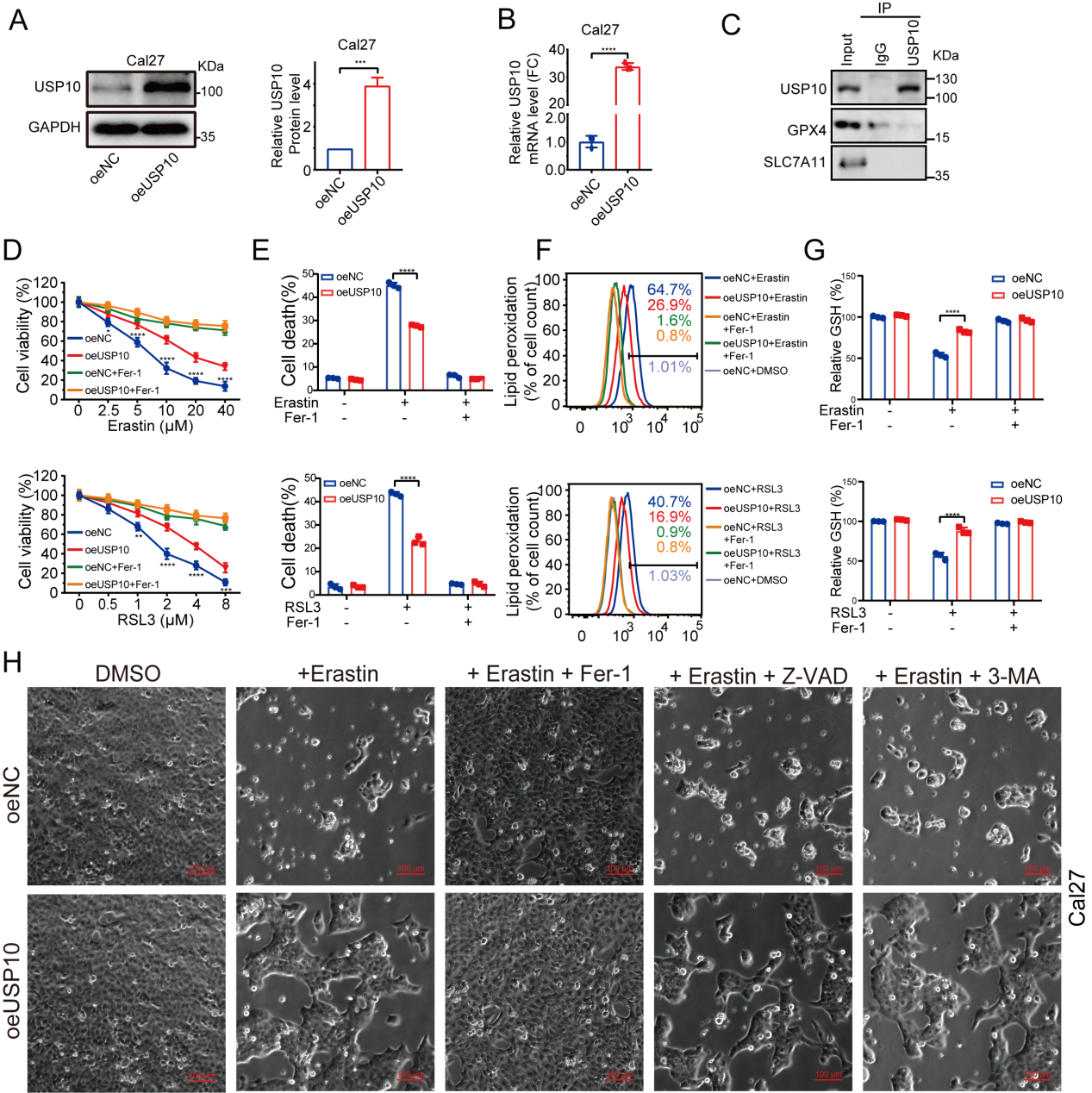


**Figure S6.** USP10 overexpression inhibits HNSCC ferroptosis sensitivity.

A-B) Western blot analysis of USP10 protein level (A) and qPCR analysis of *USP10* mRNA level (B) in Cal27 USP10 overexpression (oeUSP10) cells. oeNC means cells overexpressed nonsense gene sequence. C) Interaction of endogenous USP10 with SLC7A11 and GPX4 in Fadu cells was detected by co-immunoprecipitation. D) oeUSP10 Cal27 cells were treated with different concentrations of Erastin or RSL3 for 24  h, with or without the addition of Fer-1(1 μM), and then cell viability was assessed by CCK8. E-G) oeUSP10 Cal27 cells were treated with Erastin (10 μM) or RSL3 (2 μM), and cell death (E), lipid peroxidation levels (F) and GSH levels (G) were detected. H) Representative phase-contrast images of Cal27 cells were treated with Erastin (10 μM), Erastin (10 μM) and Fer-1 (1 μM), Erastin (10 μM) and Z-VAD-FMK (Z-VAD, 5 μM), or Erastin (10 μM) and 3-methylademine (3-MA, 25 μM). Scale bar, 100μM. All data are representative of at least three independent experiments. Data are presented as mean  ±  SD, n  =  3. *p* value was determined by two-tailed unpaired Student’s t test (A, B) and 2-way ANOVA (C-F); ns, not significant (*p* > 0.05); *, *p* < 0.05; **, *p* < 0.01; ***, *p* < 0.001; ****, *p* < 0.0001.


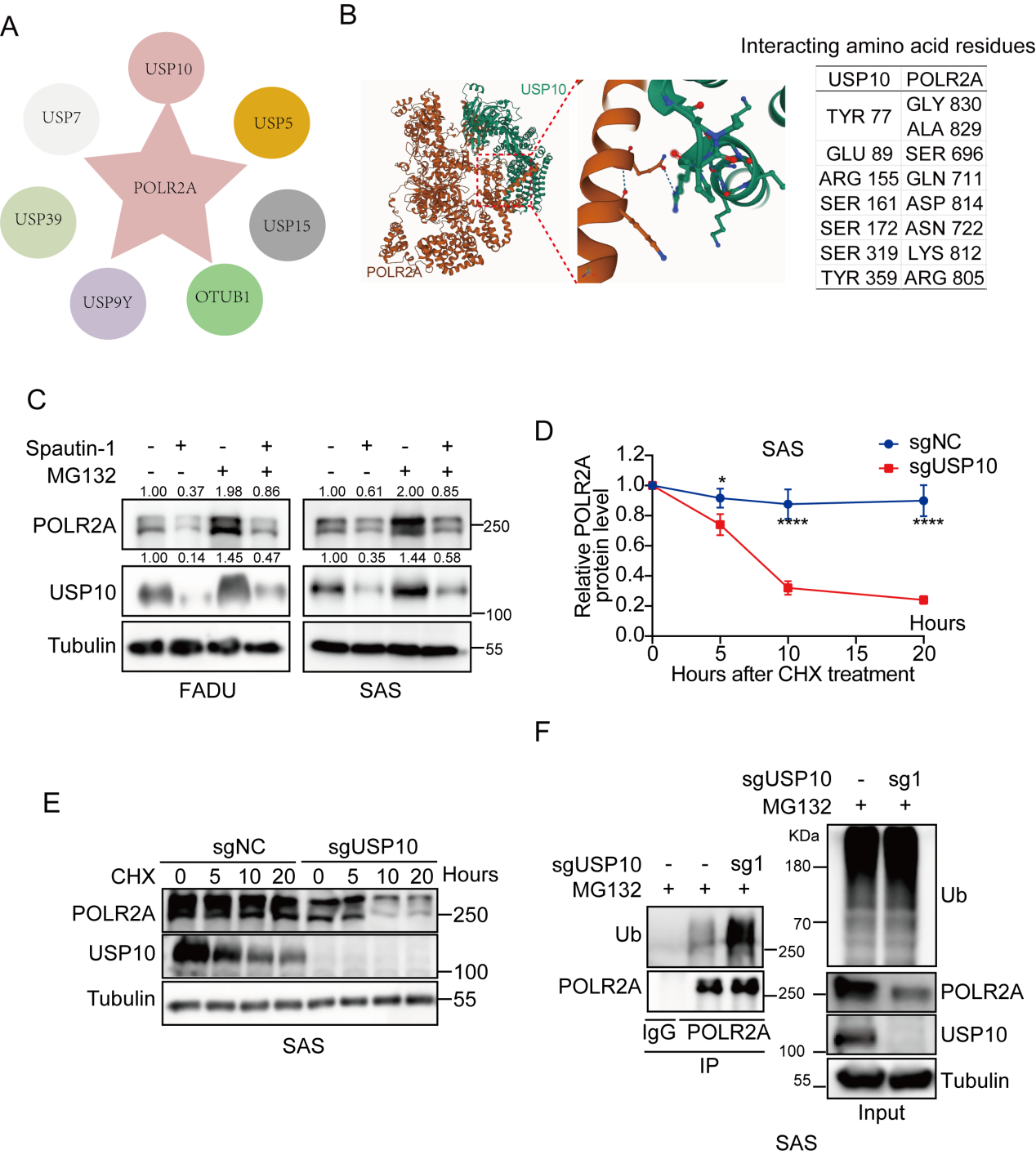


**Figure S7.** USP10 Stabilizes POLR2A through its deubiquitinase activity.

A) Potential POLR2A-interacting DUBs were identified by mass spectrometry analysis. B) Schematic representation of the predicted interaction regions between USP10 and POLR2A using AlphaFold3 (left), and we identified that the USP10 amino acid residues at positions 77 (TYR), 89 (GLU), 155 (ARG), 161 (SER), 172 (SER), 319 (SER), and 359 (TYR) are predicted to interact with POLR2A (right). C) Fadu and SAS cells pre-incubated with Saputin-1 (40  μM) for 24 h, then treated with DMSO or MG132 (10 μM) for 12 h before harvesting. Then USP10 and POLR2A protein levels were detected by immunoblot. D-E) USP10 depleted Fadu cells were treated with cycloheximide (CHX) (50 μg/ml) for different times. USP10 protein level was detected by immunoblot. F) USP10 depleted SAS transfected with HA-Ub were treated with MG132 (10 μM) for 12h before harvesting, and cell lysates subjected to IP with His magnetic beads, followed by IB with indicated antibodies. All data are representative of at least three independent experiments. Data are presented as mean  ±  SD; n  =  3. *p* value was determined by two-way ANOVA; *, *p* < 0.05; ****, *p* < 0.0001.


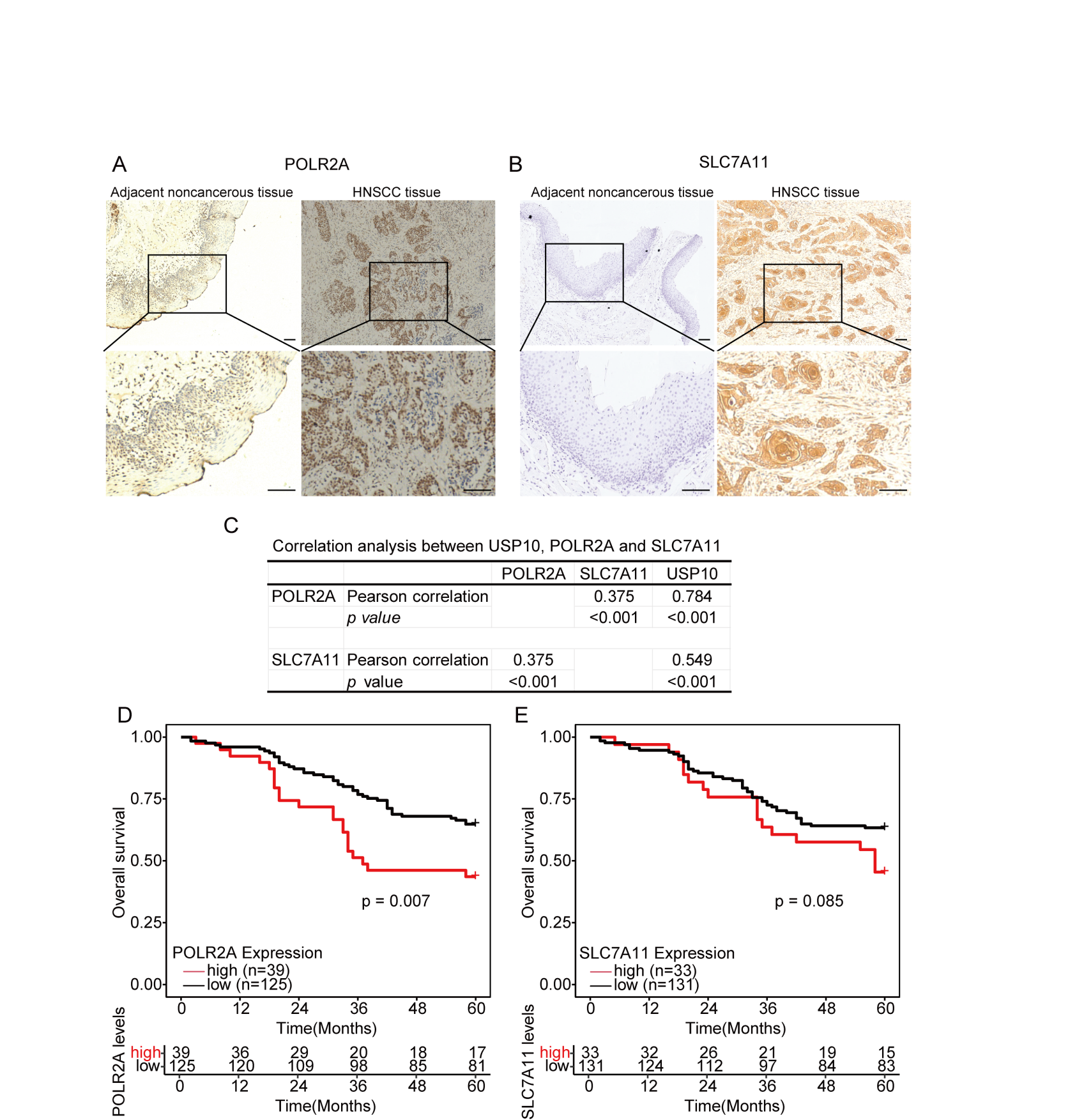


**Figure S8.** Clinical significance of POLR2A and SLC7A11 in HNSCC patients.

A and B) Representative immunohistochemical staining for POLR2A (A) and SLC7A11 (B) in HNSCC tissues (n = 167). Scale bars, 100 μm. C) Pearson correlation analysis confirmed a positive correlation between USP10 and POLR2A and SLC7A11. D-E) Kaplan-Meier analysis of overall survival in all patients according to protein level of POLR2A (D) and SLC7A11 (E). The log-rank test was used to calculate the *p* value.
