# Supplementary material for: USP10 Inhibits Ferroptosis via Deubiquinating POLR2A in Head and Neck Squamous Cell Carcinoma
Source: Adv Sci (Weinh). 2025 Jul 2;12(36):e12271. doi: 10.1002/advs.202412271 (PMC12462914; doi:10.1002/advs.202412271)
Supplement: Supplementary file 2 — Supporting Information [file ADVS-12-e12271-s002.zip › Table S2.docx]

| **Table S2.** Clinical information of HNSCC patients in public online datasets of TCGA, GSE42743 and GSE41613 | | | |
| --- | --- | --- | --- |
|  | **TCGA** | **GSE41613** | **GSE42743** |
| **Age** |  |  |  |
| ≥58 | 317 (63.3%) | 50 (51.6%) | 61 (59.2%) |
| <58 | 184 (36.7%) | 47 (48.5%) | 42 (40.8%) |
| **Gender** |  |  |  |
| Male | 368 (73.5%) | 66 (68.0%) | 79 (76.7%) |
| Female | 133 (26.5%) | 31 (32.0%) | 24 (23.3%) |
| **T classification** |  |  |  |
| T0-T2 | 179 (35.7%) | - | 55 (53.4%) |
| T3-T4 | 267 (53.3%) | - | 48 (46.6%) |
| TX | 33 (6.6%) | - | - |
| Not reported | 22 (0.4%) | - | - |
| **Clinical stage** |  |  |  |
| I-II | 94 (18.8%) | 41 (42.3%) | - |
| III-IV | 339 (67.6%) | 56 (57.7%) | - |
| Not reported | 68 (13.6%) | - | - |
| **Metastasis status** |  |  |  |
| N0 | 170 (33.9%) | - | 41 (39.8%) |
| N+ | 238 (47.5%) | - | 62 (60.2%) |
| NX | 69 (13.8%) | - | - |
| Not reported | 24 (4.8%) | - | - |
| **Treatment** |  |  |  |
| Radiation Therapy (RT) | 179 (35.7%) | - | - |
| Chemotherapy (CT) + RT | 76(15.2%) | - | - |
| Surgery (S) | 1(0.2%) | - | 38 (36.9%) |
| RT + S | 113(22.6%) | - | 58 (56.3%) |
| CT + S |  | - | 1 (1.0%) |
| CT + RT + S | 89(17.8%) | - | 5 (4.9%) |
| RT + S + others | 2(0.4%) | - | - |
| CT + RT+ others | 1(0.2%) | - | - |
| CT + RT+S+others | 12(2.4%) | - | - |
| Not reported | 28(5.6%) | - | 1 (1.0%) |
| **Primary tumor site** |  |  |  |
| Larynx | 121 (24.2%) | - | - |
| Tongue | 149 (29.7%) | - | - |
| Oral cavity | 228 (45.5%) | - | 100 (97.1%) |
| Oropharynx | - | - | 3 (2.9%) |
| Other | 3 (0.6%) | - | - |
| **Smoking** |  |  |  |
| Current | - | - | 40 (38.8%) |
| Former | - | - | 42 (40.8%) |
| Never | - | - | 21 (20.4%) |
