# Supplementary material for: USP10 Inhibits Ferroptosis via Deubiquinating POLR2A in Head and Neck Squamous Cell Carcinoma
Source: Adv Sci (Weinh). 2025 Jul 2;12(36):e12271. doi: 10.1002/advs.202412271 (PMC12462914; doi:10.1002/advs.202412271)
Supplement: Supplementary file 2 — Supporting Information [file ADVS-12-e12271-s002.zip › Table S7. Correlations analysis of POLR2A and SLC7A11.docx]

| **Table S7.** Correlations between the expression of POLR2A and SLC7A11 protein and clinicopathological parameters in patients with HNSCC | | | | | | | | | | |
| --- | --- | --- | --- | --- | --- | --- | --- | --- | --- | --- |
|  |  | **POLR2A** | | | |  | **SLC7A11** | | | |
| **Parameters** | **Number** | **Low** | **High** | **χ^2^** | ***p*-value^*^** |  | **Low** | **High** | **χ^2^** | ***p*-value^*^** |
| Age |  |  |  | 0.01 | 0.859 |  |  |  | 1.256 | 0.253 |
| <58 | 77 | 57 | 20 |  |  |  | 58 | 19 |  |  |
| ≥58 | 90 | 69 | 21 |  |  |  | 74 | 16 |  |  |
| Gender |  |  |  | 0.371 | 0.253 |  |  |  | 0.677 | 0.194 |
| Male | 163 | 124 | 39 |  |  |  | 130 | 33 |  |  |
| Female | 4 | 2 | 2 |  |  |  | 2 | 2 |  |  |
| Alcohol consumption |  |  |  | 4.83 | **0.021** |  |  |  | 0.391 | 0.430 |
| Yes | 83 | 56 | 27 |  |  |  | 63 | 20 |  |  |
| No | 65 | 55 | 10 |  |  |  | 53 | 12 |  |  |
| Unknown | 19 | 15 | 4 |  |  |  | 16 | 3 |  |  |
| Smoking history |  |  |  | 0.421 | 0.442 |  |  |  | 1.25 | 0.222 |
| Yes | 90 | 71 | 19 |  |  |  | 75 | 15 |  |  |
| No | 63 | 46 | 17 |  |  |  | 47 | 16 |  |  |
| Unknown | 14 | 9 | 5 |  |  |  | 10 | 4 |  |  |
| Histological grade |  |  |  | 0.001 | 1 |  |  |  | 0.08 | 0.694 |
| G1 | 106 | 80 | 26 |  |  |  | 85 | 21 |  |  |
| G2+G3 | 61 | 46 | 15 |  |  |  | 47 | 14 |  |  |
| Primary tumor site |  |  |  | 1.81 | 0.128 |  |  |  | 0.955 | 0.307 |
| Glottic | 114 | 90 | 24 |  |  |  | 93 | 21 |  |  |
| Others | 53 | 36 | 17 |  |  |  | 39 | 14 |  |  |
| T classification |  |  |  | 3.059 | 0.072 |  |  |  | 4.76 | **0.022** |
| T1+T2 | 87 | 71 | 16 |  |  |  | 75 | 12 |  |  |
| T3+T4 | 80 | 55 | 25 |  |  |  | 57 | 23 |  |  |
| Clinical stage |  |  |  | **9.498** | **0.001** |  |  |  | **5.298** | **0.013** |
| I-II | 69 | 61 | 8 |  |  |  | 61 | 8 |  |  |
| III-IV | 98 | 65 | 33 |  |  |  | 71 | 27 |  |  |
| Metastasis status^#^ |  |  |  | **23.967** | **<.001** |  |  |  | **5.95** | **0.01** |
| N0 | 108 | 95 | 13 |  |  |  | 92 | 16 |  |  |
| N+ | 59 | 31 | 28 |  |  |  | 40 | 19 |  |  |
| **NOTE:** *, *p* ≤ 0.05 was considered to be statistically significance.  ^#^, Metastasis status indicates lymph node and/or distant metastasis. | | | | | | | | | | |
