# Supplementary material for: USP10 Inhibits Ferroptosis via Deubiquinating POLR2A in Head and Neck Squamous Cell Carcinoma
Source: Adv Sci (Weinh). 2025 Jul 2;12(36):e12271. doi: 10.1002/advs.202412271 (PMC12462914; doi:10.1002/advs.202412271)
Supplement: Supplementary file 2 — Supporting Information [file ADVS-12-e12271-s002.zip › Table S9. Primers used in the study.docx]

| **Name** | **Sequence** |
| --- | --- |
| *SLC7A11* | Forward: 5′-GGTCCATTACCAGCTTTTGTACG-3′ |
|  | Reverse: 5′-AATGTAGCGTCCAAATGCCAG-3′ |
| *GPX4* | Forward: 5′-GAGGCAAGACCGAAGTAAACTAC-3′ |
|  | Reverse: 5′-CCGAACTGGTTACACGGGAA-3′ |
| *FSP1* | Forward: 5′-AGTAGTGGGGATAGACCTGAAGA-3′ |
|  | Reverse: 5′-CCACCACGATGAACCGTGA-3′ |
| *DHODH* | Forward: 5′-GTTCTGGGCCATAAATTCCGA-3′ |
|  | Reverse: 5′-TCTGGGTCTAGGGTTTCCTTC-3′ |
| *POLR2A* | Forward: 5′-GAGAGCGTTGAGTTCCAGAACC-3′ |
|  | Reverse: 5′-TGGATGTGTGCGTTGCTCAGCA-3′ |
| *GAPDH* | Forward: 5′-TCCAAAATCAAGTGGGGCGA-3′ |
|  | Reverse: 5′-AGTAGAGGCAGGGATGATGT-3′ |
| *SLC7A11* promoter | Forward: 5′-TGAGTGGTGGCCTCTATCTTAC-3′ |
|  | Reverse: 5′-CCCCACTCAGATAGGAAACTG-3′ |
| *USP10* | Forward: AAATGCCACCGAACCTATCGGC |
|  | Reverse: CAGCCATTCAGACCGATCTGGA |
| *USP10* knockout validation | Forward: TTGTTCAGCGTAAGTAAAGGACG |
|  | Reverse: GACCAAAATCAGCCCCGGG |

**Table S9.** Primers used in the study
