# Supplementary material for: USP10 Inhibits Ferroptosis via Deubiquinating POLR2A in Head and Neck Squamous Cell Carcinoma
Source: Adv Sci (Weinh). 2025 Jul 2;12(36):e12271. doi: 10.1002/advs.202412271 (PMC12462914; doi:10.1002/advs.202412271)
Supplement: Supplementary file 2 — Supporting Information [file ADVS-12-e12271-s002.zip › Table S3. Correlations analysis of USP10 protein.doc]

| **Table S3.** Correlations between the expression of USP10 protein and clinicopathological parameters in patients with HNSCC | | | | | |
| --- | --- | --- | --- | --- | --- |
| **USP10 expression** | | | | | |
| **Parameters Number Low High χ^2^ value *p*-value^*^** | | | | | |
| Age |  |  |  |  |  |
| <58 | 79 | 31 | 48 | 2.044 | 0.153 |
| ≥58 | 88 | 44 | 44 |  |  |
| Gender |  |  |  |  |  |
| Male | 163 | 73 | 90 | 0.043 | 0.836 |
| Female | 4 | 2 | 2 |  |  |
| Alcohol consumption |  |  |  |  |  |
| Yes | 83 | 31 | 52 | 2.675 | 0.102 |
| No | 65 | 33 | 32 |  |  |
| Unknown | 19 | 11 | 8 |  |  |
| Smoking history |  |  |  | 0.936 | 0.333 |
| Yes | 90 | 40 | 50 |  |  |
| No | 63 | 33 | 30 |  |  |
| Unknown | 14 | 2 | 12 |  |  |
| Histological grade |  |  |  | 0.092 | 0.762 |
| G1 | 106 | 45 | 61 |  |  |
| G2+G3 | 61 | 30 | 31 |  |  |
| Primary tumor site |  |  |  |  |  |
| Glottic | 114 | 59 | 55 | **6.801** | **0.009** |
| Others | 53 | 16 | 37 |  |  |
| T classification |  |  |  |  |  |
| T1+T2 | 87 | 50 | 37 | **11.582** | **0.001** |
| T3+T4 | 80 | 25 | 55 |  |  |
| Clinical stage |  |  |  |  |  |
| I-II | 69 | 46 | 23 | **22.496** | **<.001** |
| III-IV | 98 | 29 | 69 |  |  |
| Metastasis status^#^ |  |  |  |  |  |
| N0 | 108 | 65 | 43 | **28.829** | **<.001** |
| N+ | 59 | 10 | 49 |  |  |
| **Note:** *, *p* ≤ .05 was considered to be statistically significance.  ^#^, Metastasis status indicates lymph node and/or distant metastasis. | | | | | |
