# Supplementary material for: USP10 Inhibits Ferroptosis via Deubiquinating POLR2A in Head and Neck Squamous Cell Carcinoma
Source: Adv Sci (Weinh). 2025 Jul 2;12(36):e12271. doi: 10.1002/advs.202412271 (PMC12462914; doi:10.1002/advs.202412271)
Supplement: Supplementary file 2 — Supporting Information [file ADVS-12-e12271-s002.zip › Table S4. The overall survival Cox regression analysis.doc]

| **Table S4．** The overall survival Cox regression analysis | | |
| --- | --- | --- |
|  | **Relative risk (95% CI)** | ***p-*value** |
| **Univariate**  Gender  Age  Smoking history  Alcohol intake  Histological grade  Primary tumor site  T classification  Clinical stage  Metastasis  USP10 expression  **Multivariate**  Metastasis  USP10 expression | 0.805 (0.197-3.294)  0.855 (0.619-1.507)  0.918 (0.563-1.496)  1.691 (0.999-2.862)  1.115 (0.552-2.252)  0.437 (0.269-0.710)  1.897 (1.156-3.111)  3.785 (2.060-6.954)  4.355 (2.633-7.202)  3.954(2.188-7.145)  2.761 (1.282-5.949)  1.999 (1.049-3.809) | 0.763  0.524  0.732  **0.05**  0.762  **0.001**  **0.011**  **< 0.001**  **< 0.001**  **< 0.001**  **0.010**  **0.035** |
| **Note:** *p*-values in bold were statistically significant.  **Abbreviation:** 95% CI, 95% confidence interval | | |
